# Comparison of electropenetrography waveform libraries for *Nipaecoccus viridis* (Hemiptera: Pseudococcidae) using different tethering materials and monitor settings

**DOI:** 10.1093/jisesa/ieaf063

**Published:** 2025-06-27

**Authors:** Emilie P Demard, Elaine A Backus, Lauren M Diepenbrock

**Affiliations:** Department of Entomology and Nematology, Citrus Research and Education Center, University of Florida, Lake Alfred, FL, USA; USDA Agricultural Research Service, San Joaquin Valley Agricultural Sciences Center, Parlier, CA, USA; Department of Entomology and Nematology, Citrus Research and Education Center, University of Florida, Lake Alfred, FL, USA

**Keywords:** hibiscus mealybug, electropenetrography, probing behavior, citrus, tethering method

## Abstract

The hibiscus mealybug, *Nipaecoccus viridis* (Newstead) is a phloem-feeding pest that was first documented in Florida citrus orchards in 2019. Feeding causes fruit and leaf deformation due to cellular changes in host plant tissues. Field assays suggest that systemic insecticides can disrupt the probing behavior of this phloem feeder. However, the mechanisms involved are poorly understood. The objective of this study was to investigate the feeding interactions of second–third instar *N. viridis* on Volkamer lemon trees (*Citrus volkameriana*) using AC–DC Electropenetrography. Since preliminary recordings failed to distinguish phloem salivation from phloem ingestion waveforms, the effects of 3 tethering materials to improve waveform resolution were tested: thick gold wire (25 µm diameter), fine gold wire (12.5 µm diameter), and Wollaston platinum wire (2.5 µm diameter). In addition, a combination of 3 different input resistances (Ri) (amplifier sensitivities) and substrate voltages; 10^9^ Ω with 250 mV; 10^10^ Ω with 100 mV; and 10^13^ Ω with 0 mV were compared to create a waveform library. The best-quality signal was obtained with the thick gold wire (25 µm diameter) at Ri 10^10^ Ω using the loop method of wiring. Wollaston platinum wire impeded nymphal movement, causing increased nonprobing duration and increased time from the start of the recording to the first phloem salivation. Biological interpretations of waveforms are discussed in light of fruit and leaf distortion. Results from this study will allow future work to compare effectiveness of insecticides to prevent such damage.

## Introduction

The hibiscus mealybug, *Nipaecoccus viridis* (Newstead) (Hemiptera: Pseudococcidae), was first reported in Florida in 2009 on dodder (*Cuscuta exaltata* Engelm.) (Convolvulaceae: Solanales) (Stocks and Hodges 2010) and then identified on citrus in 2019 (Diepenbrock and Ahmed 2020). *Nipaecoccus viridis* is polyphagous, infesting a broad range of hosts from cultural crops to ornamental plants (Sharaf and Meyerdirk 1987, García-Morales et al. 2016). On citrus, adults and nymphs feed on the phloem of leaves, flowers, fruits, and branches (Olabiyi et al. 2023a). Feeding causes fruit distortions, curling of leaves, increased leaf drop, and premature fruit abortion (Sharaf and Meyerdirk 1987, Levi-Zada et al. 2019). Like other sternorrhynchan hemipterans, mealybugs produce a sugary secretion referred to as honeydew, which results in sooty mold development on leaves and fruit (Franco et al. 2004). Heavy infestations can result in branch dieback, and ultimately death of young citrus trees (Diepenbrock and Ahmed 2020).

The hibiscus mealybug preferentially feeds in protected locations such as leaf axils, fruit calyx, and branch intersections (Diepenbrock and Ahmed 2020). Because of its cryptic feeding behavior, small size, and clumped distribution, it is difficult to detect the pest in time to prevent damage. In addition, the insect is covered with a protective waxy coating which limits contact and penetration of insecticide droplets leading to reduced efficacy (Sharaf and Meyerdirk 1987, Franco et al. 2004). Field studies suggest that foliar and drench application of systemic insecticides may disrupt the ability of the adult insects to feed, likely by impacting stylet probing behaviors of the mealybug and can provide residual effects up to 4 wk post-treatment (Middleton and Diepenbrock 2022, Demard and Diepenbrock 2023). Yet, the mechanisms of *N. viridis* probing behavior and how insecticides may interfere with it are still unknown.

Because different definitions are encountered in the literature regarding the terminology for feeding behavior, it is important to clearly define the terms used here in our paper. We define “feeding” as all behaviors on the plant that culminate in ingesting plant fluid (ie including probing, penetration, and ingestion)  (Backus 2000) . “Stylet probing” or “stylet penetration” are synonymous and refer to all types of stylet insertions regardless of duration or purpose. Ingestion is defined as the uptake of fluids from plants into the alimentary canal of the insect.

Electropenetrography (EPG) is a successful method used to study and quantify the probing behaviors of hemipterans. It was first developed by McLean and Kinsey (1964) and has been modified and improved in the past decades (Tjallingii 1978, Backus and Bennett 2009). EPG monitors measure changes in resistance and biopotentials in a closed circuit including the insect and the plant (Walker 2000, Backus et al. 2019). When the insect probes the plant, the fluid in the stylets conducts the electrical signal to the EPG where it is amplified and displayed as waveforms on a computer screen. Different voltage patterns called “waveforms” represent different steps of the probing behavior such as stylet movements, salivation, and ingestion. Waveforms are identified and characterized based on voltage level, frequency, duration, and electrical origins (defined below) (Tjallingii 1978, Walker 2000).

To define biological meanings of waveforms, researchers must fulfill the “triangle of correlations” associating waveforms with insects’ stylet activities and locations in the host plant via histological studies (like stylectomy followed by sectioning and examination of plant tissue), honeydew production, or direct observation of the stylets in transparent artificial diets (Backus 1994, Walker 2000). However, correlation studies can be time-consuming, tedious, and expensive due to the specialized techniques and technologies required. *Nipaecoccus viridis* is an invasive species, and the need to quickly understand its probing behavior to limit damage at fruit onset and effect population control makes biological studies undesirable.

An alternative way to understand the biological meaning of waveforms is to create and interpret a waveform library, possible with the third generation AC–DC 4-channel monitor (Backus and Bennett 2009), whose flexible settings can be adjusted for different arthropods (Backus and Shih 2020). In particular, the head-stage amplifier has selectable input resistance (*R*_i_) levels from 10^6^ to 10^10^ plus 10^13^ Ω allowing the user to match the *R*_i_ with the inherent resistance of the arthropod (*R*_a_) regardless of its body size. Thus, a waveform library is defined as a selection of waveform appearances when the *R*_i_ level and substrate (excitation) voltages are changed (Backus and Shih 2020). The change in *R*_i_ levels during a recording allows the experimenter to determine the mixture of electrical origins represented by each waveform (resistance [R] component and electromotive force [emf] component). R is the physical resistance to the passage of ionized fluids moving through the stylets and the electrical conductivity of these fluids. The emf components, also called biopotentials, originate from breakage of plant cell membranes by the stylets or streaming potentials caused by rapid fluid movements through narrow capillary tubes like the stylet canals (Walker 2000, Backus et al. 2016). Because the biological meanings of R and emf components are known, researchers can hypothesize their waveforms’ meanings based on changes in appearance for R and emf components.

Each insect has a unique inherence resistance (*R*_a_) depending on its body size and the diameter of the food and salivary canals in the stylets (Backus et al. 2019). Consequently, each insect has unique amplifier responsiveness curves that describe the proportions of R or emf in its waveforms when recorded at different *R*_i_ levels (Tjallingii 1978, 1988, Backus and Bennett 2009, Backus et al. 2019). The ideal emf/R mixture is obtained when the *R*_i_ selected matches the *R*_a_ of the insects recorded, at which point the R:emf ratio (in amplitude) will be 50:50 (Backus et al. 2019). Thus, all possible waveforms will be visible. For the second-to-third instars of *N. viridis*, R/emf responsiveness has not been described yet. However, we know that for most insects R-component waveforms are emphasized at low *R*_i_ levels (10^6^ to 10^7^ Ω), thus taller, while at moderate *R*_i_ levels (10^9^ to 10^10^ Ω) a mixture of R and emf is present often with emf dominating. Finally, at the highest *R*_i_ (10^13^ Ω), the waveform is exclusively due to emf only (Backus and Bennett 2009).

Since its development, EPG studies have been applied to many sternorrhynchan insects including psyllids (Serikawa et al. 2012, George et al. 2018, Ebert and Rogers 2020), thrips (Kindt et al. 2003), aphids (van Helden and Tjallingii 1993, Prado and Tjallingii 1994, Garzo et al. 2016), and whiteflies (Lei et al. 2001, Jiang and Walker 2003) but also to auchenorrynchae (Almeida and Backus 2004, Sandanayaka et al. 2013). Six papers have been published on mealybug probing behaviors using second generation DC monitors (fixed *R*_i_ of 10^9^ Ω): 2 on the cassava mealybug, *Phenacoccus manihoti* Matile-Ferrero (Hemiptera: Pseudococcidae) (Calatayud et al. 1994, 2001), 2 on the citrus mealybug, *Planococcus citri* Risso (Hemiptera: Pseudococcidae) (Cid and Fereres 2010, Obok et a. 2018), 2 on the solenopsis mealybug, *Phenacoccus solenopsis* Tinsley (Hemiptera: Pseudococcidae) (Huang et al. 2012, 2014), one each on the longtailed mealybug, *Pseudococcus longispinus* (Targioni Tozzetti) (Hemiptera: Pseudococcidae), and the obscure mealybug, *Pseudococcus viburni* (Signoret) (Hemiptera: Pseudococcidae) (Obok et al. 2018). Only Catalayud et al. (1994) investigated the waveform-behavior correlation using light microscopy.

Our original objective was to further quantify phloem salivation using AC–DC monitors, which have more flexible *R*_i_ settings, and identify probing behaviors that have not been observed with DC monitors. We hypothesize that phloem salivation (waveform E1) is correlated with the induction of tissue distortions from feeding. Indeed, numerous studies have shown that the watery saliva of aphids plays a role in the secretion of certain metabolites such as enzymes and proteins, and the induction of host responses (Tjallingii 2006, Will et al. 2007, 2009, Carolan et al. 2009, Moreno et al. 2011, Garzo et al. 2016). Consequently, reducing the time *N. viridis* spends in phloem salivation may lead to reduced plant damage. Tentative attempts were made to wire adult female mealybugs, but even when brushing the dorsal part of the insect, the dense and heavy wax secreted by the insect prevented good adhesion of the glue causing the wire to snap. Moreover, gravid female hibiscus mealybugs produce a ventral sac called ovisac which contains eggs. After 3 to 5 wk of oviposition, adult females die within the ovisac (Sharaf and Meyerdink 1987). When selecting adult females from our colony, we were unsure about how far females were in the preoviposition process and the development of their ovisacs, which may disturb their feeding behavior.

As a result, recordings were done using second-to-third instar females because these stages persist long enough (5 to 6 d duration) to complete an entire recording and do not interfere with other biological phenomena such as egg laying (Olabiyi et al. 2023b). However, preliminary AC–DC EPG recordings failed to distinguish phloem salivation (waveform E1) from phloem ingestion (waveform E2). To enhance the quality of our recordings, we tested the effect of 3 tethering materials on the probing behavior of second- and third-instar *N. viridis;* a thin gold wire of 12.5 µm diameter (sold as 0.005 in; see Materials and Methods), a thick gold wire of 25 µm diameter (sold as 0.0010 in), and a Wollaston platinum wire of 2.5 µm. We also created a waveform library at 3 different *R*_i_ levels (10^9^; 10^10^; 10^13^ Ω) that bracket the calculated *N. viridis R*_a_ (see Materials and Methods section for calculation).

The objectives of the study were to: (i) create a waveform library and propose biological meanings based on the electrical origins of the waveforms; (ii) compare the waveforms with previous studies done on mealybugs and propose modifications to their characterization, if necessary; (iii) compare the effects of tethering materials on waveform appearance and probing behavior; and (iv) identify the best tethering methods and monitor settings for future quantitative studies with *N. virdis*. Durations and counts of EPG variables depending on the tethering materials are also discussed.

## Materials and Methods

### Insect and Plant Maintenance

Hibiscus mealybugs were collected in 2019 from Highlands County, FL (latitude: 27°20′24.00″ N; longitude: −81°20′24.00″ W) and a colony was established at the Citrus Research and Education Center (CREC), Lake Alfred, FL (Olabiyi et al. 2023b). The colony was set up and maintain by individually transferring crawlers and nymphal stages collected from the field on clean plants using a sable brush. Mealybugs were reared on Volkamer lemon seedlings (*Citrus volkameriana*) of 6 mo to 1 yr old at about 25 ± 3 °C. Plants were reared in 3.9-liter black plastic containers filled with about 1.9 kg of soil (Pro-Line HydraFiber C/B, Jolly Gardener, Southern Agricultural Insecticides Inc., Palmetto, FL, USA) and fertilizer (Citrus, Avocado, and Mango 6-4-6, Sunniland Corporation, The Home Depot, Winter Haven, FL, USA).

### EPG Recordings

Recordings were done on the abaxial surface of Volkamer lemon young leaves. Young leaves were defined as soft, light green in color and about 3 to 4 wk old after bud swelling (stages V4 to V5 as classifified by Cifuentes-Arenas et al. 2018). Two, 4-channel AC–DC monitors (Backus and Bennett 2009) custom built by William Bennett (EPG Equipment Co., Otterville, Missouri) were used, applying DC + voltage with 20× fixed amplification in the head-stage amplifier. To reduce electronic noise, the plant–insect system was placed in a Faraday cage built with an aluminum frame covered with pure copper screen (16 × 16 mesh = 0.15 mm wire spaced 1.58 mm apart) (Supplementary Fig. S1.A). A ring stand was placed in the Faraday cage to support the electronics (Supplementary Fig. S1.B). We also set up a voltage regulator (Furman P-1800 AR Power conditioner, 15 A, 120V, Sweetwater) plugged into the power supply on each monitor to reduce the “floor noise”. A 35-mm diameter plastic Petri dish was placed in the ring stand to provide a flat surface to which to attach the leaf, for the insect to feed upon and prevent plant movement from breaking the electrical circuit. The leaf was held abaxial surface-up with double-sided tape to the outside flat surface of the Petri dish (Supplementary Fig. S1.C). Light was provided by overhead fluorescent lights (24:0 h L:D photoperiod). Room temperature was maintained at about 26 ± 3 °C.

Initially mealybugs were moved from the plant colony to a Petri dish with a sable brush by first poking their abdomen to stop them from feeding before transferring them. Mealybugs were then starved for a short period of time (2 to 4 h) in the Petri dish sealed with parafilm before being wired. However, we noticed that these insects took longer than anticipated to initiate probing during the recordings. Therefore, we increased the starvation period to 24 h from the time they were removed from the colony until they were placed on the plants. When using the thick gold wire (2.5 cm L × 25.4 µm D; Sigma Cohn Corp., Vernon, New York), a small loop at the end of a wire was made to increase surface contact with the thorax cuticle and obtain better conductivity (Cervantes and Backus 2018). When using the fine gold wire (2.5 cm L × 12 µm D; Sigma Cohn Corp., Vernon, New York), the *en pointe* method was used because the wire was too brittle to make a loop (Cervantes and Backus 2018). The tip of the wire was then dipped into the silver glue to attach it to the mealybug thorax. The glue was made by mixing white glue (Elmer’s liquid school glue), water, and silver flake (size 8 to 10 µm; Inframat Advanced Materials, Manchester, Connecticut) at a ratio of 1:1:1 vol:vol:wt. For the Wollaston platinum wire, mealybugs were attached by the thorax by following the protocol described in the Supplementary Material of Chesnais and Mauck (2018). The end of the platinum wire (2.5 µm D × 0.5 cm L; Wollaston process wire; Sigmund Cohn Corp., Mt. Vernon, New York, USA) was dipped into nitric acid at 45% and allowed to dry for a couple of minutes. The tip of platinum wire was then dipped into water-based silver glue (Supplementary Fig. S2).

Body lengths of 7 second- and third-instar female mealybugs were measured with a Leica stereomicroscope using a reticle eyepiece with a scale in mm (Supplementary Table S1). Length averaged 1.76 mm and body width 1.23 mm (Supplementary Fig. S3). We used the stylet food and salivary canal diameters of other mealybug species available in the literature (Kaydan et al. 2015, Alliaume et al. 2018) to calculate the mealybug resistance (*R*_a_) at which the signal will contain 50:50 R:emf if the *R*_i_ is set to match. The stylet diameters were averaged between the 2 papers and a new formula for calculating *R*_a_ was used (E. A. Backus, unpublished data, ms. in prep). We obtained a theoretical *R*_a_ of 1.17 × 10^12^ Ω for second- and third-instar females. Because *R*_i_ of 10^11^ and 10^12^ Ω are not available on the amplifier of an AC–DC monitor, we chose *R*_i_ levels that bracketed 10^12^ Ω, therefore 10^9^, 10^10^, and 10^13^ Ω *R*_i_.

For all 3 tethering methods, the other end of the wire was attached to a copper wire (23 mm L × 0.48 mm D) with silver glue. The wire was inserted into the unit’s head-stage amplifier. To complete the electrical circuit, a copper electrode (10 cm L × 2 mm D) was inserted into the soil of the potted plant. Before the start of each recording, plants were watered to reach saturation and increase electrical contact (Walker 2000).

Recordings were started before placing the mealybugs on the plants and the material was touch-tested by gently touching the copper wire with a finger when the *R*_i_ level was set at 10^6^ Ω (and grounding the body by holding the side of the Faraday cage). If a distinct peak was observed on the recording software, then the amplifier was considered functional. The insect was observed for ~2 s to make sure it was touching the plant’s surface and could move freely despite the wire attached to it. All recordings started at the “np” (nonprobing) behavior and lasted for 24 h. Pre- and postrectification signals were checked for signal inversion caused by the rectifier. If the signal was inverted, the offset knob was adjusted to revert it (Backus and Shih 2020). Baseline voltages were determined using the prerectification signal, but figures were derived from postrectification signals, for less noise.

Data were acquired, stored, and displayed with WinDaq Pro + data acquisition software using a DI 710 AD converter (Dataq Instruments Inc., Akron, Ohio) at a sampling rate of 100 Hz per channel. WinDaq Waveform Browser (also Dataq) was used to visualize, measure, and manually annotate the waveforms. The waveforms were inspected for recurring patterns and annotated following aphid standards; nonprobing (np), potential drop (pd), mesophyll pathway (C), xylem ingestion (G), stylet derailment (F), phloem salivation (E1), phloem ingestion (E2), and extracellular salivation (E1e). A new waveform (see Results section) was also annotated and called waveform S for likely breakage of mesophyll cell membranes.

## Experimental Design

### Waveform library

Eight insects were recorded each day on 2 AC–DC monitors. Three input resistance levels and voltage substrates (*V*_s_) combinations were tested: (i) 10^9^ Ω with *V*_s_ = 250 mV; (ii) 10^10^ Ω with *V*_s_ = 100 mV, and (iii) 10^13^ Ω with *V*_s_ = 0 V. These choices were to enable each *R*_i_ level to enhance either R (moderate *R*_i_ + high *V*_s_), R/emf mixture (higher *R*_i_ + low *V*_s_), or pure emf (highest *R*_i_ + no *V*_s_). The tethering method was blocked by monitor (4 channels with the same tethering materials) and rotated every day, such as:

- Day 1: Monitor 1 = fine gold wire vs Monitor 2 = thick gold wire;
- Day 2: Monitor 1 = platinum wire vs Monitor 2 = fine gold wire;
- Day 3: Monitor 1 = thick gold wire vs Monitor 2 = platinum wire.

Two types of recordings were carried out for the waveform library; one set was done by switching *R*_i_ levels during a recording every 2 to 3 h. Recordings started either from 10^9^ Ω and we increased *R*_i_ levels gradually up to 10^13^ Ω or they started at 10^13^ Ω and we decreased *R*_i_ level down to 10^9^ Ω. For the second set, *R*_i_ level was kept constant for the entire duration of the recording. For each monitor, 3 channels (ie insects) were set with 1 of the 3 *R*_i_ (ie 10^9^, 10^10^, 10^13^ Ω) and the fourth one was a repetition of one of the *R*_i_ levels. We rotated the *R*_i_ levels tested on each head amplifier every day as well as the repeated *R*_i_ to get approximately the same among of recordings per *R*_i_ level.

#### Quantitative Data

Based on the waveform appearance obtained with the experimental design described above, we determined that the setting 10^10^*R*_i_ with 100 mV allowed us to see the most fine-structure detail in the waveforms. Specifically, it was easier to identify and distinguish the transition from waveform E1 (phloem salivation) to E2 (phloem ingestion), which is of interest in our study. As a result, we conducted the rest of the recordings using this *R*_i_ level and substrate voltage combination. The tethering method was blocked by monitor (4 channels with the same tethering materials) and rotated every day as explained in the previous paragraph. We obtained a total of 61 recordings (19 for Wollaston platinum wire, 20 for thin gold wire, 22 for thick gold wire). Because stylet derailment (waveform F) and extracellular salivation (E1e) were both very rare events compared to other probing behaviors (<5 recordings for a total of 61 recordings analyzed) we excluded the variables correlated with these waveforms from the analysis. The new waveform S was not included in the analysis as it was not present in aphid behavior and was sporadic (present in 12 recordings). The FileManipW2 and Error Checker programs (available at https://crec.ifas.ufl.edu/extension/epg/data-analysis/) were used to clean and format the data and to check for errors in the WinDaq notepads when annotating the waveforms. The Trimmer program was used to remove any artificially interrupted events as suggested by Ebert et al. (2018). Data were analyzed using the Ebert 1.0 program which was originally created to analyze aphid’s waveforms; thus we simplified it and only kept the waveforms identified for *N. viridis*. Trimmer and Ebert 1.0 programs are also available online at https://crec.ifas.ufl.edu/extension/epg/data-analysis/. All analyses were conducted using proc glimmix using SAS version 9.4 (SAS/STAT 15.1; SAS Institute, Cary, NC). Distribution was adjusted depending on the variables; for count data, a negative binomial distribution was used when overdispersion was detected (*χ*^2^/df > 1) while for duration data, a normal distribution was used. For the duration of some events, time was changed from seconds to hours or minutes to fit the value ranges.

## Results

### Waveform Descriptions and Variability in Appearance

We followed the waveform labeling published in previous EPG studies on mealybug (Calatayud et al. 1994, Cid and Fereres 2010, Huang et al. 2012, Obok et al. 2018), which are based on aphid probing behaviors (Tjallingii 1978). We also identified several differences in waveform appearance and identification that we discuss in the following paragraphs. Second- and third-instar hibiscus mealybugs produced 8 families of EPG waveforms: A, B, C, pd, E, G, S, and F, as well as 2 types and 1 subtype within family E: E1, E2, and E1e, respectively (Table 1). These waveforms were characterized using frequencies, relative amplitude, and electrical origin (R/emf) (Table 2). Change in *R*_i_ levels was used to test R and emf components of those waveforms.

**Table 1.** Summary of the main characteristics and correlations of *Nipaecoccus viridis* nymphal stage waveforms and comparison with other mealybug species on various hosts

Mealybugs waveform characteristics					Correlation		
*N. viridis*	*P. manihoti*	*Pl. citri*	*P. solenopsis*	*Ps. longispinus* *Pl. citri* *Ps. viburni*	Stylet position in plant tissue	Activity	Voltage level
Lemon	Cassava	Grapevine	Cotton	Cacao			
Demard et al. (2025)	Catalayud et al. (1994)	Cid and Fereres (2010)	Huang et al. (2012)	Obok et al. (2018)			
np	np	np	np	np	nonprobing	Walking	…
A	A	A	A	A	Parenchyma	Secretion of salivary sheath and stylet pathway	Extracellular
B			B	B			Extracellular
C	C CI/CII/CIII	C	C	C			Extracellular
pd pre-pd/pd1/pd2	pd/E1	pd pre-pd/pd1/pd2	pd pd1/pd2	pd	Sieve element	Cell puncture	Intracellular
E1e	H	H	E1e	E1e	Unknown	Extracellular salivation	Extracellular
E1 E11/E12	E1 E1/E2I	E1 E1/E21	E1 E11/E12	E1	Sieve element	Phloem Salivation	Intracellular
E2	E2II	E22/E23	E2	E2	Sieve element	Phloem Ingestion	Intracellular
G			G	G	Xylem	Xylem Ingestion	Extracellular
F			F	F	Cell walls of all tissues	Stylet derailment/ Penetration difficulties	Extracellular
S	…	…	…	…	Parenchyma	Breakage of the mesophyll cells membrane	Intracellular

**Table 2.** Characteristics of AC–DC waveforms generated by *Nipaecoccus viridis* nymphal stage waveforms on *Citrus volkameriana*

EPG waveform		Voltage level	Electrical origin^a^	Frequency (Hz)	Relative amplitude (%)	Optimal *R*_*i*_ levels (Ω)
np						
A		Extracellular	R	5 to 10	90% to 100%	10^9^ to 10^10^
B		Extracellular	R	0.05 to 0.8	70% to 90%	10^9^ to 10^10^
C		Extracellular	R/emf	Irregular	15% to 30%	10^9^ to 10^10^
pd	pd1	Intracellular	emf	1to 2	…	10^10^ to 10^13^
	pd2		R/emf	6 to 8	…	10^9^ to 10^10^
E1e		Extracellular	R/emf	1 to 2	10% to 20%	…
E1	E11	Intracellular	R/emf	0.5 to 1.5	5% to 10%	10^10^ to 10^13^
	E12		R/emf	0.5 to 4		10^9^ to 10^10^
E2		Intracellular	emf	Irregular	5%	10^10^ to 10^13^
G		Extracellular	R/emf	3 to 4	50% to 100%	10^9^ to 10^10^
F		Extracellular	R	7 to 10	20% to 40%	…
S		Intracellular	R/emf	Irregular	…	10^10^

^a^where R = resistance, and emf = electromotive force.

**Fig. 1.**
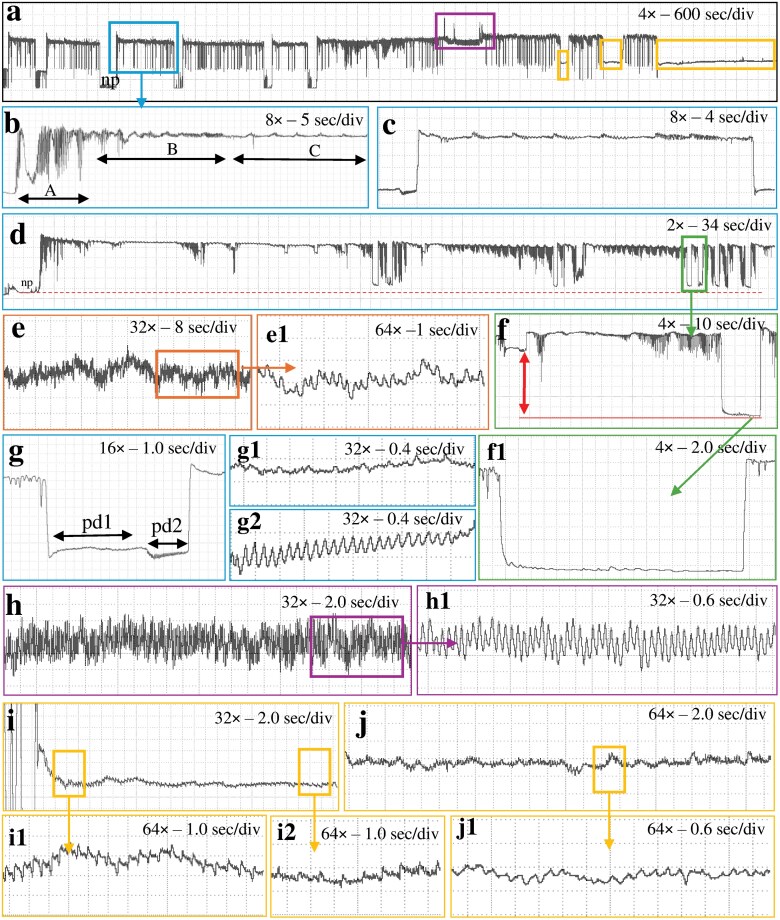
Waveforms obtained with *Nipaecoccus viridis* on *Citrus volkameriana* using AC–DC monitor with a platinum wire at *R*_i_ 10^10^ Ω and 100 mV substrate voltage. a) Overview of a recording; b) beginning of a probe showing waveform A followed by waveforms B and C; c) view of waveform C which corresponds to intercellular stylet pathway between 2 intracellular punctures; d) pathway phase composed of waveforms C, pd and S; e) waveform F corresponding to stylet derailment; e1) fine structure of waveform F; f) waveform S showing lower voltage level than pd; g) waveform pd corresponding to intracellular puncture; g1/g2) pd subphases identified as pd1 and pd2 respectively; f1) close view of waveform S corresponding to stylet withdraw at the extracellular level; h) waveform G corresponding to xylem ingestion; h1) fine structure of waveform G; i) waveform E1 corresponding to phloem salivation; i1/i2) E1 subphases identified as E11 and E12, respectively; j) waveform E2 corresponding to phloem ingestion; j1) fine structure of waveform E2.

**Fig. 2.**
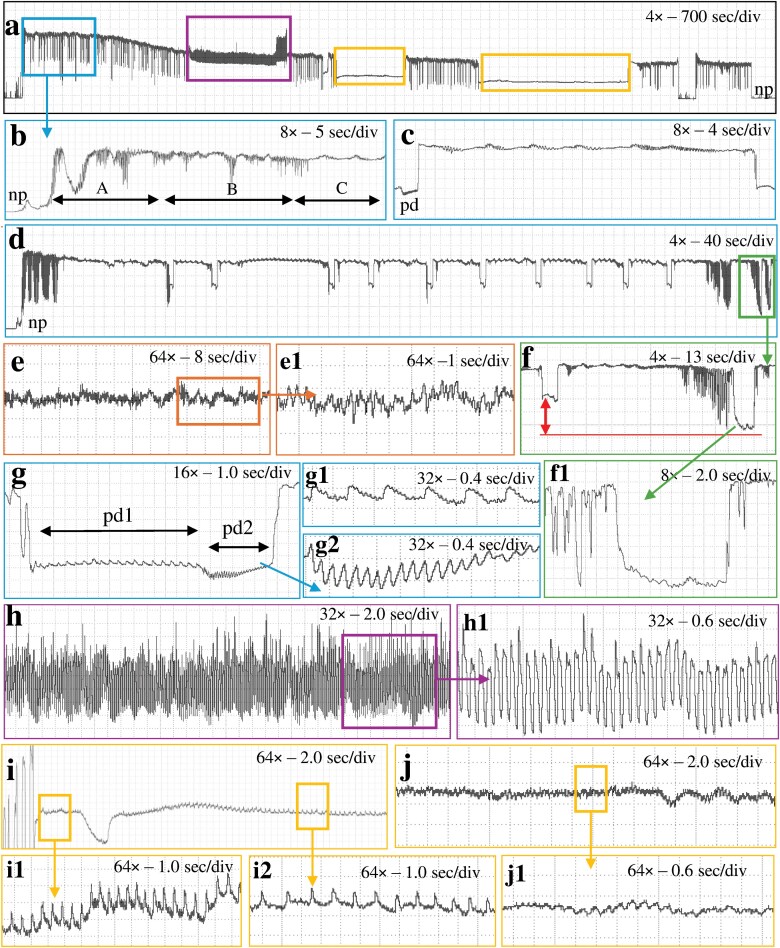
Waveforms obtained with *Nipaecoccus viridis* on *Citrus volkameriana* using AC–DC monitor with a fine gold wire at *R*_i_ 10^10^ Ω and 100 mV substrate voltage. a) Overview of a recording; b) beginning of a probe showing waveform A followed by waveforms B and C; c) view of waveform C which corresponds to intercellular stylet pathway between 2 intracellular punctures; d) pathway phase composed of waveforms C, pd and S; e) waveform F corresponding to stylet derailment; e1) fine structure of waveform F; f) waveform S showing lower voltage level than pd; g) waveform pd corresponding to intracellular puncture; g1/g2) pd subphases identified as pd1 and pd2, respectively; f1) close view of waveform S corresponding to stylet withdraw at the extracellular level; h) waveform G corresponding to xylem ingestion; h1) fine structure of waveform G; i) waveform E1 corresponding to phloem salivation; i1/i2) E1 subphases identified as E11 and E12, respectively; j) waveform E2 corresponding to phloem ingestion; j1) fine structure of waveform E2.

**Fig. 3.**
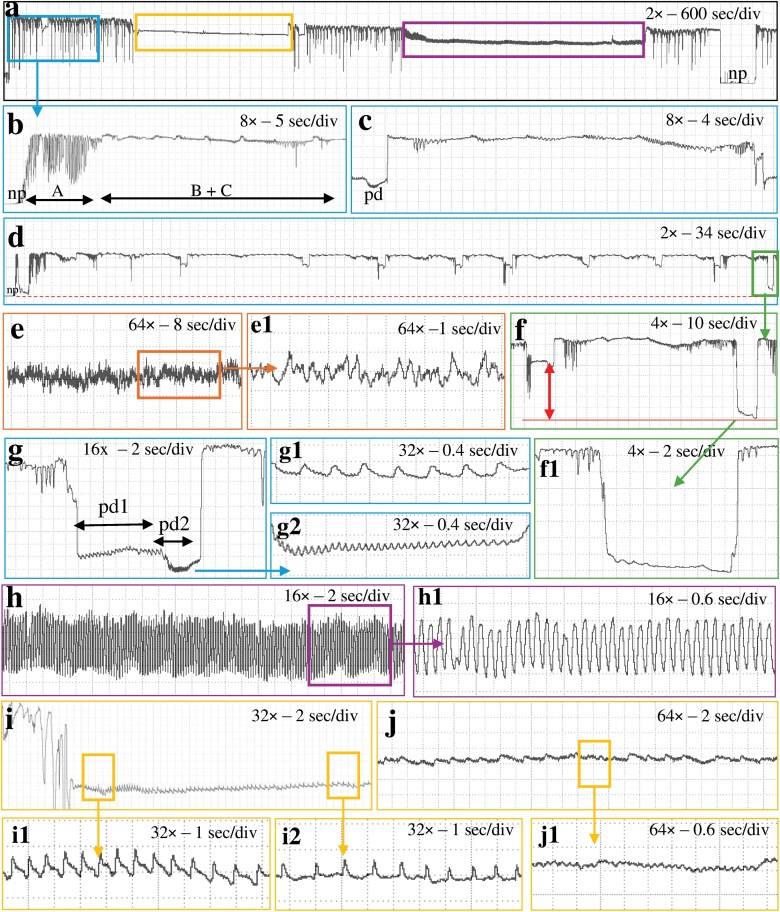
Waveforms obtained with *Nipaecoccus viridis* on *Citrus volkameriana* using AC–DC monitor with a thick gold wire at *R*_i_ 10^10^ Ω and 100 mV substrate voltage. a) Overview of a recording; b) beginning of a probe showing waveform A followed by waveforms B and C; c) view of waveform C which corresponds to intercellular stylet pathway between 2 intracellular punctures; d) pathway phase composed of waveforms C, pd and S; e) waveform F corresponding to stylet derailment; e1) fine structure of waveform F; f) waveform S showing lower voltage level than pd; g) waveform pd corresponding to intracellular puncture; g1/g2) pd subphases identified as pd1 and pd2 respectively; f1) close view of waveform S corresponding to stylet withdraw at the extracellular level; h) waveform G corresponding to xylem ingestion; h1) fine structure of waveform G; i) waveform E1 corresponding to phloem salivation; i1/i2) E1 subphases identified as E11 and E12, respectively; j) waveform E2 corresponding to phloem ingestion; j1) fine structure of waveform E2.

Regardless of the *R*_i_ levels and the tethering materials, waveforms were both monophasic and biphasic (Backus and Shih 2020), ie most waveforms were positively oriented and occurred above the baseline (monophasic) except waveform S and pd, which could go either below the baseline (biphasic) similar to those of aphids or go positive (Figs 1–3).

#### Waveform np

Baselines were always negative. Np was composed of 2 main components: a flat part with no voltage variability most likely corresponding to standing still and a part including highly variable peaks, smaller in amplitude than probes (stylet insertion) probably corresponding to labial dabbing, antennating, or walking. In previous studies, these baselines were separated into 2 different waveforms “np” for walking/labial dabbing and “z” for standing still (Youn et al. 2011). We did not differentiate these behaviors as they are not the main focus of our study.

#### Waveforms A and B

These waveforms only appear at the beginning of a probe (stylet insertion). Waveform A showed the highest relative amplitude (90% to 100%) composed of many irregular spikes of variable frequency range (5 to 10 Hz). Waveform B had a slightly lower relative amplitude (70% to 90%), and lower frequency range (0.1 to 0.8 Hz) than waveform A. Waveform B always followed waveform A and preceded waveform C. It is a transition between A and C with the relative amplitude decreasing over time making it sometimes hard to distinguish from waveform C (Fig. 3). Peaks of waveforms A and B increased when *R*_i_ decreased and applied voltage increased, indicating that peaks are R-dominated, while the underlying wave is emf-dominated (Supplementary Fig. S4).

#### Waveform C

**Fig. 4.**
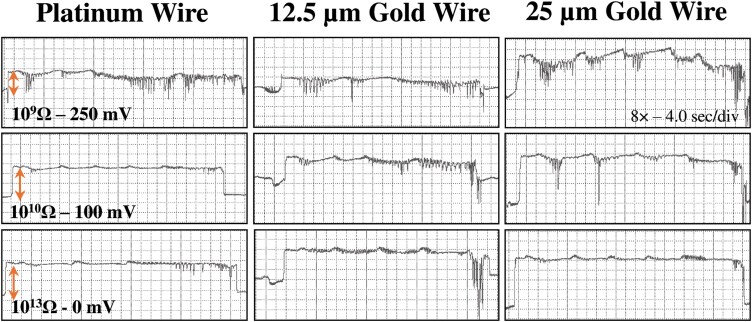
Comparison of C waveforms generated by *Nipaecoccus viridis* on *Citrus volkameriana* using 3 different tethering methods and 3 *R*_i_ level-substrate voltage combinations. Recordings were performed for 24 h with DC applied signal and *R*_i_ levels were switched during a recording, thus the waveforms displayed in the same column come from the same insect. WinDaq gain and time scale are shown in the top right box for 10^9^ Ω; the same gains and scales were used for the other graphs. The vertical arrow on the left inside represents the change in voltage rise with increased *R*_i_ levels.

Waveform C had a moderate relative amplitude (15% to 30%) with irregular frequencies. While C looks the same as C in aphids, mealybug C also somewhat resembles waveform D in psyllids (Bonani et al. 2010, Pearson et al. 2014). Waveform C was characterized by a series of medium-amplitude, slightly rounded, or triangular waves that slowly decreased in amplitude and sometimes evolved into downward square peaks at the end of the waveform (Supplementary Fig. S5). The relative amplitude of these waves was higher at *R*_i_ 10^9^ and decreased when *R*_i_ increased, supporting that R was the dominant component. However, at *R*_i_ 10^9^ Ω, waveform C occurred at a lower voltage level, making it difficult to distinguish from pd, while at *R*_i_ 10^13^ Ω waveform C exhibited a high rise compared to pd (Fig. 4). This supports the notion that the rise in voltage is emf-dominated.

#### Waveform pd

**Fig. 5.**
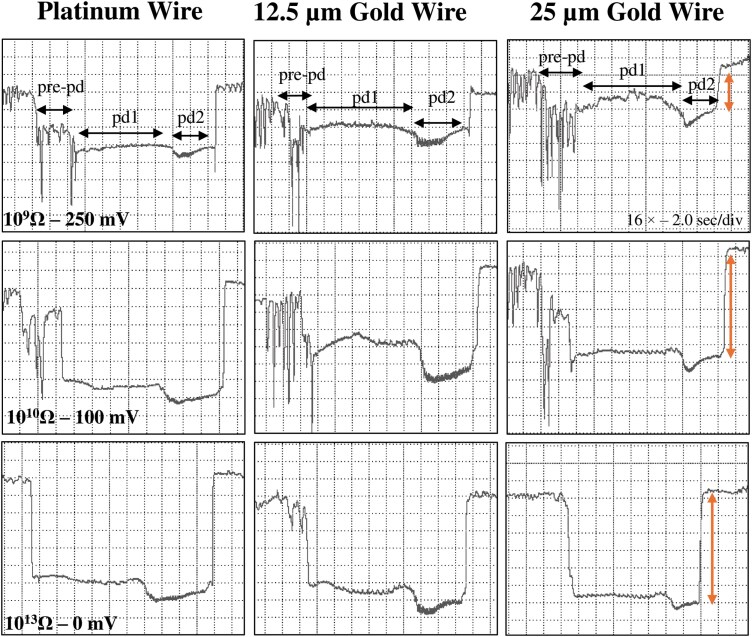
Comparison of pd waveforms generated by *Nipaecoccus viridis* on *Citrus volkameriana* using 3 different tethering methods and 3 *R*_i_ level and substrate voltage combinations. Recordings were performed for 24 h with DC applied signal and *R*_i_ levels were switched during a recording, thus the waveforms displayed in the same column come from the same insect (ie 3 columns = 3 insects). WinDaq gain and time scale are shown in the top right box for 10^9^ Ω; the same gains and scales were used for the other graphs. The vertical arrow on the right inside represents the change in voltage drop with increased *R*_i_ levels.

Waveform pd could be slightly negative (−0.04 to −0.4 V) to positive (0.1 to 4 V). The pd was divided into 3 subphases/subtypes: pre-pd, pd1, and pd2. Pre-pd consisted of a strong drop in voltage followed by hump(s) made of irregular short falls of potentials. Pre-pd was clearly distinguishable at *R*_i_ 10^9^ Ω and almost disappeared at *R*_i_ 10^13^ Ω suggesting that pre-pd is mainly composed of R components. The pd1 was made of rounded waves of low frequencies (1 to 2 Hz) whereas pd2 was composed of sharp peaks with higher frequencies (6 to 8 Hz) (Figs 1 to 3. g1/g2). Pd1 starts with a small drop of potential after pre-pd. Waveform pd1 is made of rounded waves which were more prominent as *R*_i_ levels increased, suggesting that pd1 is dominated by emf (Supplementary Fig. S6). Pd2 appeared always at a lower voltage than pd1. Peaks of pd2 have higher amplitude at low *R*_i_ levels but were still visible at *R*_i_ 10^13^ Ω, supporting a mix of R and emf components. Waveform pd2 ended with an increase in potential to reach the extracellular voltage level. The voltage drop observed in pd was higher as *R*_i_ levels increased, supporting that this drop is pure emf (Fig. 5).

#### Waveform G

This waveform occurs at high amplitude (50% to 100%) and constant frequency (3 to 4 Hz), with rounded peaks and sinus waves looking like rounded valleys (Figs 1 to 3. h/h1). Peaks appeared more pointy at low *R*_i_ (10^9^ Ω) and more rounded at high *R*_i_ (10^13^ Ω) indicating that the peaks were R-dominated but the underlying waves were emf-dominated. Amplitude did not change at different *R*_i_ levels, supporting mixed R and emf components.

#### Waveform E1

**Fig. 6.**
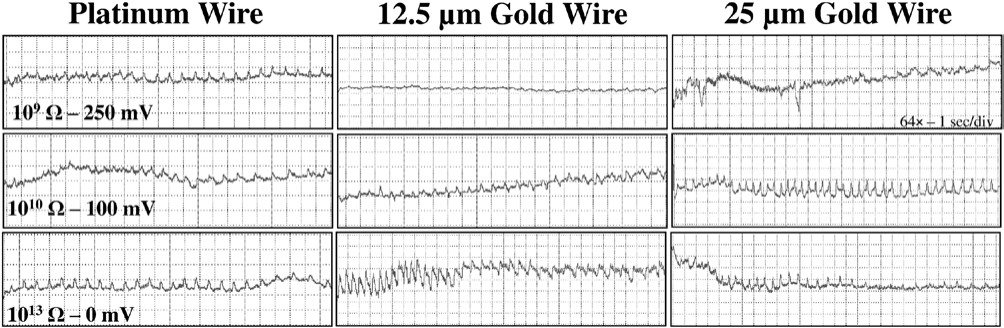
Comparison of E1 waveforms generated by *Nipaecoccus viridis* on *Citrus volkameriana* using 3 different tethering methods and 3 *R*_i_ level and substrate voltage combinations. Most of the time, it was not possible to record phloem salivation several times in one recording since mealybugs initiated the first phloem contact late in the recording. Each box represents a different insect (ie 9 boxes = 9 insects). WinDaq gain and time scale are shown in the right top box for 10^9^ Ω; the same gains and scales were used for the other graphs.

E1 was a triangle wave with regular frequency. E1 always started after waveform C with a short voltage drop. This waveform was composed of peaks followed by small spikelets of similar appearance. We divided E1 into 2 subtypes: E11 had sharp triangular peaks (Figs 1 to 3, i1) and E12 rounded peaks with longer waves (Figs 1 to 3. i2). E1 peaks are emf-dominated as they are more visible at high *R*_i_ while E1 waves were more difficult to distinguish at high Ri supporting that they were R-dominated (Fig. 6).

#### Waveform E2

**Fig. 7.**
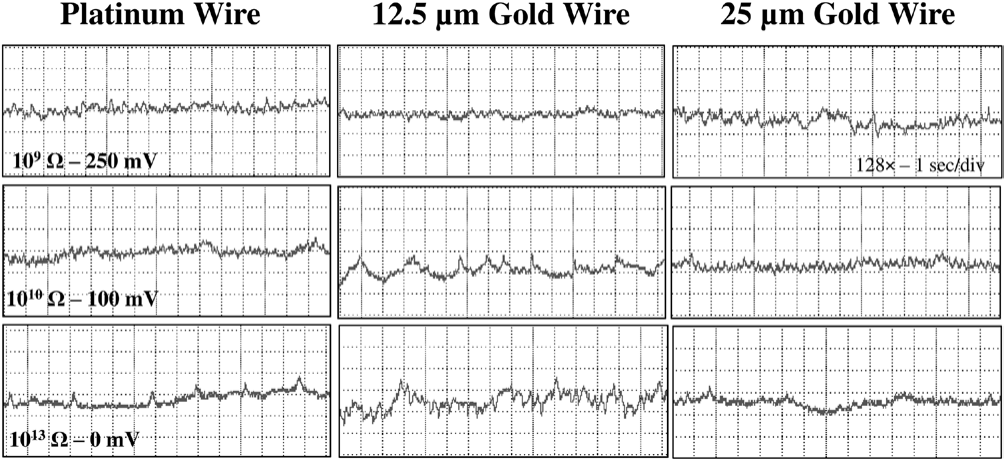
Comparison of E2 waveforms generated by *Nipaecoccus viridis* on *Citrus volkameriana* using 3 different tethering methods and 3 *R*_i_ level and substrate voltage combinations. Most of the time, it was not possible to record phloem salivation several times in one recording since mealybugs initiated the first phloem contact late in the recording. Each box represents a different insect (ie 9 boxes = 9 insects). WinDaq gain and time scale are shown in the right top box for 10^9^ Ω; the same gains and scales were used for the other graphs.

This waveform consisted of waves composed of tiny spikelets with low relative amplitude (<5%) and it always preceded E1. The spikelets forming the waves were more visible at *R*_i_ 10^9^ Ω and less prominent at *R*_i_ 10^13^ Ω suggesting an R-dominated component ( Fig. 7). The characteristic downward peaks observed in other sternorrhynchans (aphids, psyllids) and described in other mealybug studies were not observed except for one recording performed with 12 µm gold wire at *R*_i_ 10^10^ (Supplementary Fig. S7).

#### Waveform S

S is similar in appearance to pd with a bigger voltage drop than pd (see Figs 1 to 3. f) but not as low as baseline (nonprobing). Waveform S can have a positive voltage (0.3 to 1 V) or a negative voltage (−0.1 to −1 V). In contrast to pd, waveform S did not have any distinct subphase/subtype with regular patterns. This waveform was highly variable in appearance; sometimes it appeared flat similar to waveform np and sometimes it showed small waves of irregular shapes, amplitudes, and frequencies. Waveform S was present in 12 recordings and occurred between one to 14 times in each recording. Therefore, we considered it significant enough to describe and propose a name for this behavior, although its biological meaning is uncertain.

#### Waveform E1e

**Fig. 8.**
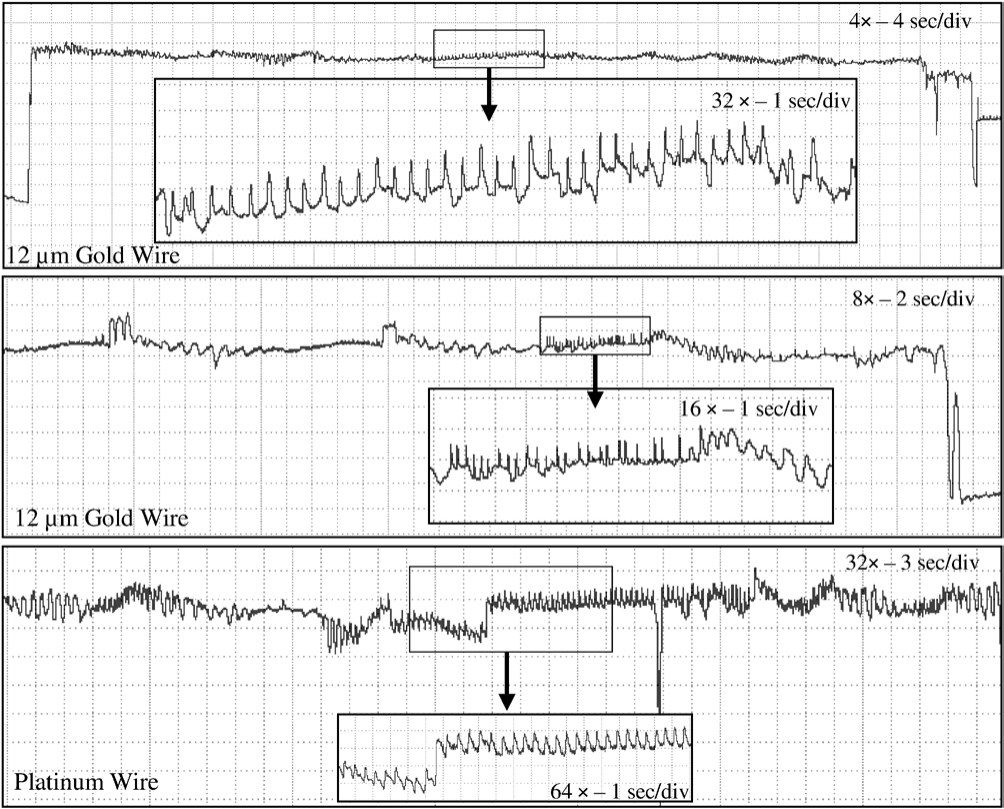
Examples of E1e waveform generated by *Nipaecoccus viridis* on *Citrus volkameriana* at *R*_i_ 10^10^ Ω and 100 mV substrate voltage. WinDaq gain and time scale are shown in the right top corners.

Waveform E1e was similar to waveform E1 but occurred at the extracellular level during waveform C. E1e appearance was quite variable in shape depending on the insect. Some recordings showed upward pointed peaks slightly variable in amplitude gathered into groups of 2 or 3 and separated with small downward peaks, while other recordings showed rounded peaks occurring at a regular frequency (1 to 2 Hz) (Fig. 8). All E1e waveforms were reported at *R*_i_ 10^10^ Ω and lasted between 8 and 60 s except for one recording for which E1e lasted 2 h and was recorded at *R*_i_ 10^9^ Ω. This finding supports that E1e is primarily R-dominated. E1e waveform was identified in only 5 recordings but occurred using all 3 tethering materials tested. Because of the low occurrence of waveforms E1e, we were unable to compare its appearance at different *R*_i_ levels and substrate voltages.

#### Waveform F

This waveform consists of regular sharp peaks with variable amplitudes and high frequencies (7 to 10 Hz) (see Figs 1 to 3. e/e1). Waveform F somewhat resembled waveform G because it occurred at the extracellular level and lasted for a long period (from 1 to 6 h). However, peaks in waveform F were irregular, they had a lower amplitude and a higher frequency than waveform G. Waveform F was detected in only 4 recordings at *R*_i_ 10^10^ Ω but reported with all 3 tethering materials, again supporting that waveform F was R-dominated. Again, because of the low occurrence of waveforms F, we did not compare its appearance at different *R*_i_ levels and substrate voltages.

### Quantitative Data Analysis

Table 3 summarizes variables that were significantly different among wire treatments. The duration of nonprobing events before first phloem salivation event was significantly higher with platinum wire than with 12 and 25 µm gold wires. Similarly, the time from start of EPG to first phloem salivation event was higher with platinum wire than with both of the gold wires. Duration of phloem salivation followed by first phloem ingestion and duration of phloem salivation followed by first sustained phloem ingestion were significantly higher with 25 µm gold wire. *Nipaecoccus viridis* performed fewer pathway events with 25 µm gold wire than with the other 2 tethering methods. Number of phloem salivation events was significantly lower with 25 µm gold wire compared to 12 µm gold wire but not from platinum wire.

**Table 3.** LSMean ± SE for behavioral parameters obtained with *Nipaecoccus viridis* feeding on *Citrus volkameriana.* Only variables that differed significantly between tethering methods are included; nonsignificant variables are presented in Supplementary Table S2

			Platinum wire		12 µm Gold wire		25 µm Gold wire					
Behavior	Variables^a^	Unit	Mean ± SE		Mean ± SE		Mean ± SE		df	*F*	*P*	Distribution
Nonprobing	Duration of np before first E1	h	5.16 ± 0.66	A	3.40 ± 0.64	AB	2.86 ± 0.61	B	2,58	3.5	0.036	Normal
Pathway	Number of C	counts	2.64 ± 0.36	A	2.61 ± 0.43	A	2.20 ± 0.31	B	2,57	3.8	0.029	Negative binomial
Salivation/ Ingestion	Number of E2	counts	2.28 ± 0.47	AB	3.80 ± 0.65	A	1.95 ± 0.39	B	2,58	5.2	0.033	Negative binomial
	Time from start of EPG to first E	h	15.33 ± 1.54	A	10.56 ± 1.46	B	10.48 ± 1.42	B	2,56	3.4	0.040	Normal
	Duration of E1 followed by first sustained E2	min	2.31 ± 0.60	B	1.44 ± 0.56	B	4.07 ± 0.60	A	2,38	5.3	0.009	Normal
	Duration of E1 followed by first E2	min	2.07 ± 0.54	B	1.66 ± 0.51	B	3.62 ± 0.51	A	2,46	4.1	0.023	Normal

Means within a column not followed by the same letter are significantly different (*P* < 0.05).

^a^A definition of each variable can be found in the Supplementary Table S3.

Thus, all nonprobing and pathway variables listed in Table 3 were shortest in duration or fewest in number with 25 µm gold wire, as was time to first E, a measure of nonprobing and pathway combined. In contrast, durations of both phloem salivation/ingestion variables were the longest with the same wire. Variables that were not statistically significant are presented in Supplementary Table S2.

## Discussion

### Impact of Tethering Methods and EPG Settings on Waveform Appearance

This is the first paper describing EPG waveforms and feeding behavior of nymphal hibiscus mealybug with any type of EPG monitor (Calatayud et al. 1994, Huang et al. 2012, 2014, Obok et al. 2018). Previous papers published on mealybugs used adults with a DC monitor at *R*_i_ 10^9^ Ω. Instead, we used the AC–DC monitor with its adjustable *R*_i_, which allows tailoring the settings to the insect’s inherent resistance (*R*_a_). Thus, in our work, we used an AC–DC monitor to compare different *R*_i_ levels to achieve a waveform library. We also compared different tethering materials for effectiveness at different *R*_i_ levels.

We were able to achieve the best balance of *R* to emf when performing EPG recordings at *R*_i_ 10^10^ Ω and 100 mV substrate voltage using thick gold wire (25 µm diameter). With this setting, we were able to improve the appearance of waveform E1 (phloem salivation); nevertheless, we were unable to observe the downward peaks characteristic of waveform E2 (phloem ingestion) as reported in previous mealybug EPG studies. We first hypothesized that the persistence of waxy secretion was impeding good electrical contact between the glue and the mealybug cuticle. Therefore, before gluing the gold wire to the insect, we tried to dip the tip of the camel brush in a solution of solvents (5% xylene and 95% ethanol) to dissolve any waxy secretion left. We recorded a dozen recordings and did not see any significant improvement in the resolution of waveform E2. Another hypothesis is the presence of residual noise in the laboratory power lines despite the setup of voltage regulators.

It was harder to identify a clear transition between phloem salivation (waveform E1) and phloem ingestion (waveform E2) in recordings using thin gold wire and Wollaston platinum. Some recordings exhibited a clear distinction between phloem salivation and ingestion, but we were not able to obtain consistent data. The tethering methods may explain this inconsistency. For thin gold wire (12.5 µm diameter) and Wollaston platinum wire, we used the *en pointe* method, which consisted of dipping the tip of the wire directly in the silver glue before placing it on the insect dorsum, while for the thick gold wire (25 µm diameter), the loop method was used. This method consists of shaping the tip of the wire into a loop before dipping it in the silver glue and then placing it on the insect (Cervantes and Backus 2018). The loop method probably increased the contact surface area between the cuticle and the glue resulting in better conductivity, explaining why better resolution was obtained with the thicker wire (Cervantes and Backus 2018). Wire thickness by itself also increased conductivity.

### Impact of Tethering Methods on Hibiscus Mealybug’s Probing Behavior

With 25 µm gold wire, the duration of phloem salivation followed by first phloem ingestion was on average 2 min and 1 min 30 s longer than with 12 µm gold wire and platinum wire, respectively. This difference is probably due to our ability to better detect waveform E1 with 25 µm gold wire resulting in a more accurate quantification of phloem salivation.

The duration of nonprobing events before first phloem contact was significantly higher with platinum wire (up to 2.4 h longer compared to 25 µm gold wire), indicating that the platinum wire might impede mealybug movement, preventing it from initiating feeding. Similarly, with platinum wire, the first phloem ingestion occurs on average 15 h after the start of the recording, which is 5 h later than with the 2 gold wires. This can be explained by the tethering flexibility; platinum wire is thinner but stiffer than the other wires used which could prevent the mealybug from moving freely and delaying the time to reach sieve elements.

The number of phloem ingestion events was significantly higher with 12 µm gold wire than with 25 µm gold wire. However, the mean duration of phloem ingestion was higher with 25 µm gold wire than 12 µm gold wire although not significantly different (3.38 and 2.37 h, respectively, Supplementary Table S2). Likewise, mean duration of the longest E2 reached 5.38 h with 25 µm gold wire whereas it lasted only 3.83 h with 12 µm gold wire. These results show that phloem ingestion events were more frequent but shorter with 12 µm gold wire than with 25 µm gold wire. This could also suggest that mealybugs interrupted phloem ingestion more often to go back to pathway phases with the 12 µm gold wire. Indeed, the number of pathway events (number of C) was significantly higher with 12 µm gold wire and platinum wire compared to 25 µm gold wire. The wiring methods used between the gold wires could explain this difference. In addition to being less conductive, the *en pointe* wiring method might be more intrusive than the loop method; the sharp tip could poke the soft-body mealybug while trying to feed.

Overall, the behavioral variables support our observations with the library waveform. We were able to better identify and separate waveform E1 from waveform E2 with 25 µm gold wire. However, we were unable to see the characteristic downward peaks of waveform E2 (ie emf components) as described in previous mealybug studies. We hypothesized that it could be due to 2 reasons; (i) the noise in the line may prevent seeing the thin structures of E2 specifically since this signal is very tiny in amplitude (proportional to the stylet diameter of the nymphs); (ii) the waxy cuticle may impede good contact with the wire preventing from seeing the downward peaks. With the 25 µm gold wire, mealybugs reached the phloem elements quicker and performed longer phloem ingestion with fewer pathway phases than with the other tethering materials.

### Biological Interpretation of Waveform and Comparison of EPG Parameters with Other Mealybugs

As previously described in other mealybugs studies, we found that waveforms A and B were dominated by R and likely correlated with initial stylet contact and secretion of salivary sheath, based on previous hemipterans correlation studies (Tjallingii and Esch 1993, Calatayud et al. 1994, Bonani et al. 2010). Fine-structure peaks of C were also R-dominated showing higher amplitude at low *R*_i_ levels. The electrical origin of this waveform and the study performed by Calatayud et al. (1994) support that waveform C is probably correlated with salivary sheath secretion in the parenchyma. Similarly to Huang et al. (2012) and Cid and Fereres (2010), we did not distinguish subphases in waveform C due to the absence of correlation studies with specific probing behavior. Nevertheless, the fine structure of C we identified in *N. viridis* (Supplementary Fig. S5) corresponds to subphase CI/CII described in Calatayud et al. (1994). The subphase CIII described by Calatayud et al. (1994) was also observed in *N. viridis* in the middle and at the end of waveform C. It is characterized by strong downward peaks which were more visible at *R*_i_ 10^9^ Ω (Fig. 4) thus indicating an R-dominated component. This component probably represents the start of the transition to pds with increased resistance as the stylet gets closer to parenchyma cell walls.

The pds observed with *N. viridis* were similar to those described by Cid and Fereres (2010) in *P. citri* due to the presence of pre-pd, which was not mentioned in other mealybug studies. These pre-pds are R-dominated and thus could represent salivation into and around the cell membrane or as the stylets encountered resistance to cell membrane breakage before reaching the intracellular level. The emf component of pd1 and pd2 represented streaming potential of fluids into the stylet. Each pd lasted on average 22 to 24 s (Supplementary Table S2), similar to the 20-s duration reported with *P. manihoti* (Calatayud et al. 1994) but shorter than the 32 s reported in *P. citri* (Cid and Fereres 2010). On the other hand, *P. solenopsis* had a much shorter pd duration with an average of 12 s (Huang et al. 2012). Depending on the tethering materials, *N. viridis* performed between 35 to 39 pds (Supplementary Table S2) per probe, which is close to the number observed in *P. citri* (43 pds per probe) (Cid and Fereres 2010). Netherveless, *N. viridis* performed noticeably more pds within a 24 h recording (from 176 to 210 depending on the wiring methods, Supplementary Table S2) than *P. citri* and *P. longispinus* which performed on average 85 and 50 pds, respectively, during the same recording duration (Obok et al. 2018).

Biological interpretation of pd in mealybugs is unclear. As mentioned by Calatayud et al. (1994) and Cid and Fereres (2010), pd1 could represent intracellular salivation and pd2 intracellular ingestion since they are similar to pd subphase II-1 and II-3 of aphids.

Because pds in mealybugs are longer than pds in aphids, they could be correlated with damage induction. *Nipaecoccus viridis* is not known to transmit pathogens, but analyzing the chemical composition of saliva and finding enzymes or proteins correlated with the host plant defense mechanisms could be the first step to understanding the role of saliva in some waveforms.

Waveform E1e may also be correlated with damage induction by *N. viridis* but at the extracellular level. Waveform E1e is also referred to as waveform H in earlier studies on mealybugs (Table 1) (Calatayud et al. 1994, Cid and Fereres 2010). We chose the nomenclature E1e as it was first identified in aphid feeding behavior (Tjallingii 1990). This waveform was rare (identified in about 5 recordings) and often occurred for only a short duration (<1 min) without a major drop in potential or change in amplitude which made it hard to detect. Additionally, the appearance of this waveform greatly varied among studies and mealybug species. The waveform E1e identified in our study was similar to the one described in Huang et al. (2012) but never found in pds as described by other authors. Nevertheless, it was very different in appearance from waveform E1e described in Obok et al. (2018) and Cid and Fereres (2010). Waveform E1e has not been correlated to specific probing behaviors in aphids and mealybugs. Calatayud et al. (2001) associated this waveform with a stress response when mealybugs are parasitized or feeding in unfavorable feeding sites.

Waveform G identified in *N. viridis* was analogous to that in other mealybugs and was correlated with xylem ingestion in previous hemipterans such as aphids and psyllids (Tjallingii 1990, Bonani et al. 2010). As described by Pearson et al. (2014), the high amplitude and R domination of G could be interpreted as the movement of cibarial muscles and the opening/closing movement of valves during ingestion. The occurrence of waveform G was rare in our recordings (13 recordings) and lasted 1.7 to 4 h on average depending on the tethering methods (Supplementary Table S3). Similar durations were found in Obok et al. (2018) with mean durations of 3 to 4 h depending on the mealybug species. Cid and Fereres (2010) reported a higher number of waveform G events in *P. citri* with durations reaching up to 17 h.

Concerning phloem salivation and ingestion, E1 and E2 distinctions were made based on previous aphid studies (Tjallingii 1990). Therefore, we defined E1 as any patterns with upward sharp peaks while E2 was defined as patterns with downward peaks. Different subphases/subtypes were identified in different mealybug species, which makes E1/E2 distinction confusing depending on the nomenclature used. In our study, waveform E1 was divided into 2 subphases E11/E12 in agreement with Huang et al. (2012) and based on the appearance of the upward peaks. Our subphase E11 in *N. viridis* was comparable to waveform E1 in *P. manihoti* and *P. citri* with sharp triangular peaks while subphase E12 was similar to E2I in *P. manihoti* and E2I in *P. citri* with more rounded peaks followed by small waves (spikelets) (Calatayud et al. 1994, Cid and Fereres 2010). Based on aphid studies, we suggest renaming the subphases E2I and E21 identified in *P. manihoti* and *P. citri*, respectively, as E12 since their appearance is closer to E1 than E2. E12 could be interpreted as the start of the transition from E1 to E2. Obok et al. (2018) is the only study that did not identify phloem subphases, which makes it easier to compare to our results. Although lacking the downward peaks, waveform E2 defined in *N. viridis* was comparable to E2II and E22/23 described in *P. manihoti* and *P. citri*, respectively (Calatayud et al. 1994, Cid and Fereres 2010). Overall, *N. viridis* spent 64% to 75% of the total probing time (ie excluding nonprobing behavior) in pathway phases (waveforms C and pd) which is greater than the time spent in phloem ingestion (18% to 28%) (Supplementary Fig. S8). This observation is quite surprising for a phloem feeder and could support the hypothesis that pd may contribute to tissue distortion as it is a frequent behavior (average of 176 to 210 pd count per recording, Supplementary Table S3).

With both gold wires, *N. viridis* took an average of 10 h (Supplementary Table S2) to initiate first phloem salivation. This duration is shorter than observed in *P. citri*, which takes 14.5 h to reach phloem on grapevine plants and 18.5 h on cacao plants (Cid and Fereres 2010, Obok et al. 2018). Due to the difference in phloem subphase identification, Cid and Fereres (2010) reported shorter phloem salivation which lasted 45 s in *P. citri* because they excluded subphase E21 (E12 in our study) from E1 duration calculation. In our study, phloem salivation lasted 4 to 9 min depending on the tethering materials (Supplementary Table S2). However, mean duration of phloem ingestion was comparable to what was reported with an average of 4.0 h per recording on a whole plant, and we obtained 2 to 3.4 h with *N. viridis* (Cid and Fereres 2010). Durations of phloem salivation and ingestion undoubtedly depend on factors such as the choice of host plants, mealybug species, and experimental setup which could also explain these differences. However, the large difference in phloem salivation duration (less than 1 min in *P. citri* but more than 5 min in *N. viridis*) could be correlated with fruit and leaf deformation in *N. viridis* although histological studies will need to confirm this hypothesis.

Waveform F was rare but, when initiated, mealybugs performed this behavior for a relatively long period (>1 h). This waveform was not described in the first 2 studies done on mealybugs (Calatayud et al. 1994, Cid and Fereres 2010). Yet, it was described in aphids (Tjallingii 1990) and reported in the last 2 studies done on mealybugs (Huang et al. 2010, Obok et al. 2018). Waveform F is interpreted as derailed stylet mechanics or stylet penetration difficulties. Due to its low occurrence, we could not interpret this behavior in relation to the tethering materials or the EPG settings.

We identified a new waveform similar to pd that we called S. Waveform S lasted 27 s on average, which was close to the duration of pds. Like waveform pd, waveform S could be positive before the voltage drop to the negative voltage level. Waveform S is different from pd by virtue of a deeper voltage drop, sometimes reaching negative voltage, and not exhibiting subphases. It was observed at all *R*_i_ levels indicating that it is composed of mix R and emf components. Because of the large, negative-going voltage drop of S, we hypothesize that this waveform represents brief plasmalemma breakage of a mesophyll cell during pathway phase; furthermore, due to the relatively long duration (compared with aphid pd’s) we hypothesize that the stylets may remain a significant time in the cell, thus perhaps barely breaking the cell membrane and move sideways through the edge of the cell, “nicking” the cell membrane. Thus, based on previous studies examining the mouthparts of the maple mealybug *Phenacoccus aceris* (Signoret) (Hemiptera: Pseudococcidae) (Alliaume et al. 2018) and studying the stylet track of *P. longispinus* in yucca leaf (Brennan et al. 2001), it is possible that mealybugs perform intracellular probing for a few seconds during otherwise intercellular probing.

While we initially hypothesized that damage induced in plants by *N. viridis* originated from phloem salivation (waveform E1), the present study of nymphal mealybug waveforms with a waveform library has suggested additional waveforms of potential interest. Particularly, extracellular salivation such as waveform E1e, or intracellular phases such as waveform pd and S could also be correlated with plant damage. Therefore, microscopy studies of stylet tracks in plant tissues as well as mouthpart and salivary gland morphology will be essential to elucidate the biological meaning of these waveforms and allow correlation with tissue distortion.

Previous studies reported how systemic insecticides can disrupt the probing behavior of the Asian citrus psyllid, *Diaphorina citri* Kuwayama (Hemiptera: Liviidae) (Serikawa et al. 2012, Miranda et al. 2016, Langdon et al. 2019, Carmo-Sousa et al. 2020). These studies showed that phloem salivation and ingestion were greatly reduced by neonicotinoid treatments, limiting pathogen inoculation into healthy plants and pathogen acquisition from infected plants. Specifically, Landgon et al. (2019) showed that soil-applied imidacloprid is taken up by the xylem and moves to the phloem where concentrations rise due to plant transpiration. Although the hibiscus mealybug spent a longer time ingesting from parenchyma than in the sieve elements, the total duration of phloem ingestion when using 25 µm gold wire reached 7 h (Supplementary Table S2), which is enough time to induce mortality. In addition, even if the hibiscus mealybug performed fewer xylem ingestion events than other mealybugs, ingestion last several hours which should be enough to kill nymphal stages.

Based on our results, foliar or drench applications of systemic insecticides could help in controlling mealybug infestation by killing nymphal stages, thus limiting female emergence and their reproduction. Indeed, preliminary results from a field study showed that thiamethoxam induced 60% to 80% mortality in hibiscus mealybug, and residues were still detected in leaves up to 14 d after application in Citrus Under Protective Screen (E. P. Demard, unpublished data, m.s. in preparation). However, our results also demonstrate that time to first probe to first phloem salivation varies from 9 to 13 h (Supplementary Table S2) which implies that systemic insecticides may fail to protect trees from tissue distortions as intra or/and extracellular salivation may have already occurred before the insect reaches the sieve elements.

### Summary and Perspectives

We showed that wiring methods and EPG settings (substrate voltage and input resistance) greatly impact waveform appearances. By standardizing the wiring methods with 25 µm wire diameter and the EPG settings with 100 mV substrate voltage and *R*_i_ level of 10^10^ Ω, we obtained better waveform resolution with both R and emf components and minimized experimental artifacts. This allowed the mealybug to better express its natural probing behaviors. Waveforms described in *N. viridis* resemble other mealybug waveforms reported in past studies, although some discrepancies were noticed with phloem salivation and ingestion. We also reported the occurrence of a new waveform (S) that needs further investigation.To protect developing fruit during spring, applications of systemic insecticides such as neonicotinoids, spirotetramat, and flupyradifurone are currently recommended when populations start to peak (ie when nymphal stages are present and ovisacs are not developed yet). Further studies need to be developed to quantify the effect of systemic insecticides in reducing fruit distortion. Specifically, the choice of active ingredient, dosage, and application methods (foliar or drench) should be compared to develop more precise recommendations for pest management programs.

## Supplementary material

Supplementary material is available at *Journal of Insect Science* online.

ieaf063_suppl_Supplementary_Figures_S1-S8_Tables_S1

ieaf063_suppl_Supplementary_Tables_S2-S3

## Notes

Disclaimer. Mention of trade names or commercial products in this publication is solely for the purpose of providing specific information and does not imply recommendation or endorsement by the U.S. Department of Agriculture. USDA is an equal opportunity provider and employer.
