# Supplementary material for: Comparison of electropenetrography waveform libraries for Nipaecoccus viridis (Hemiptera: Pseudococcidae) using different tethering materials and monitor settings
Source: J Insect Sci. 2025 Jun 27;25(3):23. doi: 10.1093/jisesa/ieaf063 (PMC12202763; doi:10.1093/jisesa/ieaf063)
Supplement: ieaf063_suppl_Supplementary_Figures_S1-S8_Tables_S1 [file ieaf063_suppl_supplementary_figures_s1-s8_tables_s1.docx]

Supplementary materials


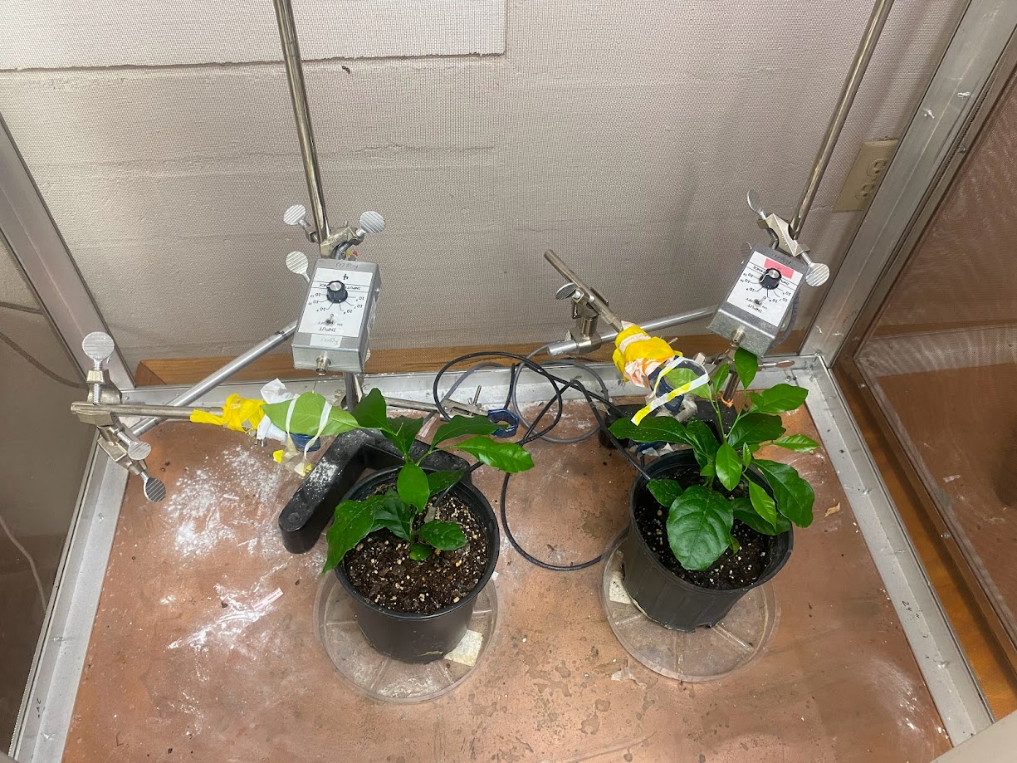

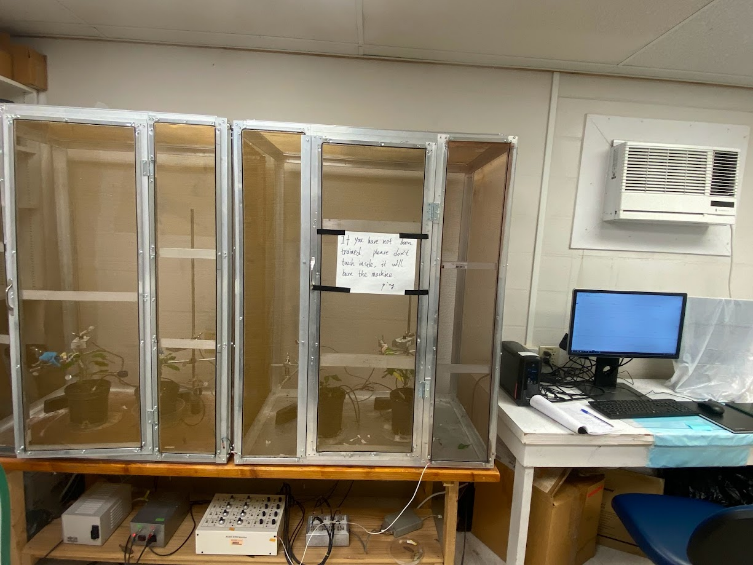


**A**

**B**

Ring stands

Head amplifiers

Soil electrodes

Faraday cage


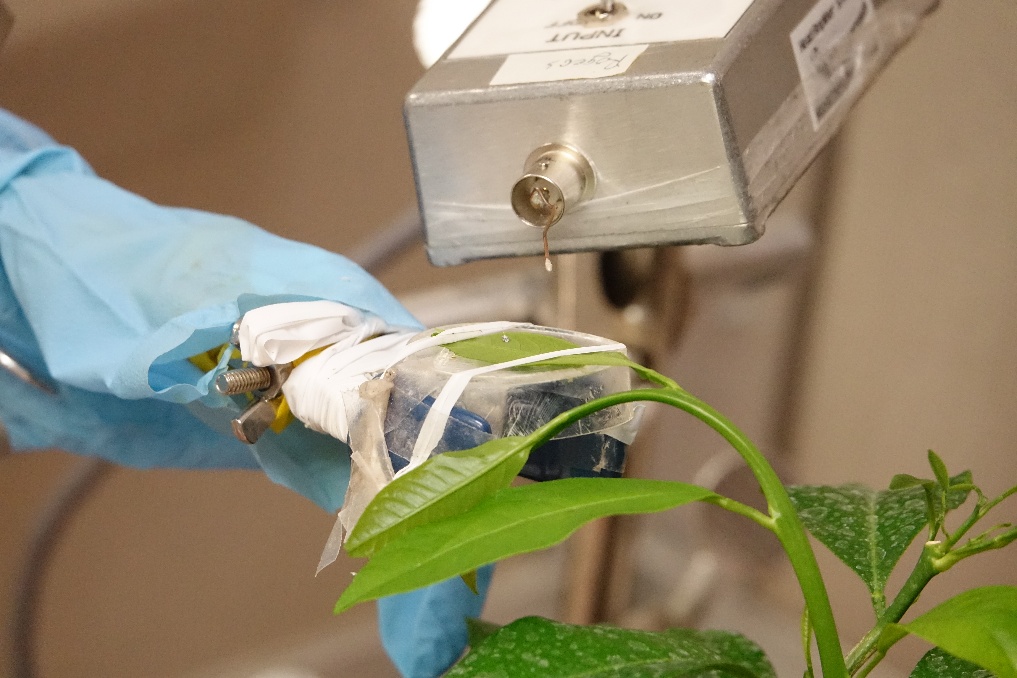


Mealybug

Petri dish

**C**

Head amp.

Fig. S1. Overall set up of the EPG system. A) Faraday cage made of copper screen; B) View of the ring stands, head amplifiers, soil electrode and plants inside the Faraday cage; C) Close view of the head amplifier with the petri dish and leaf placed upside down.


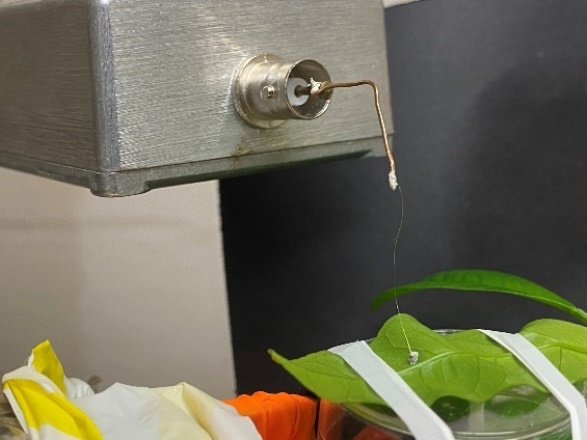

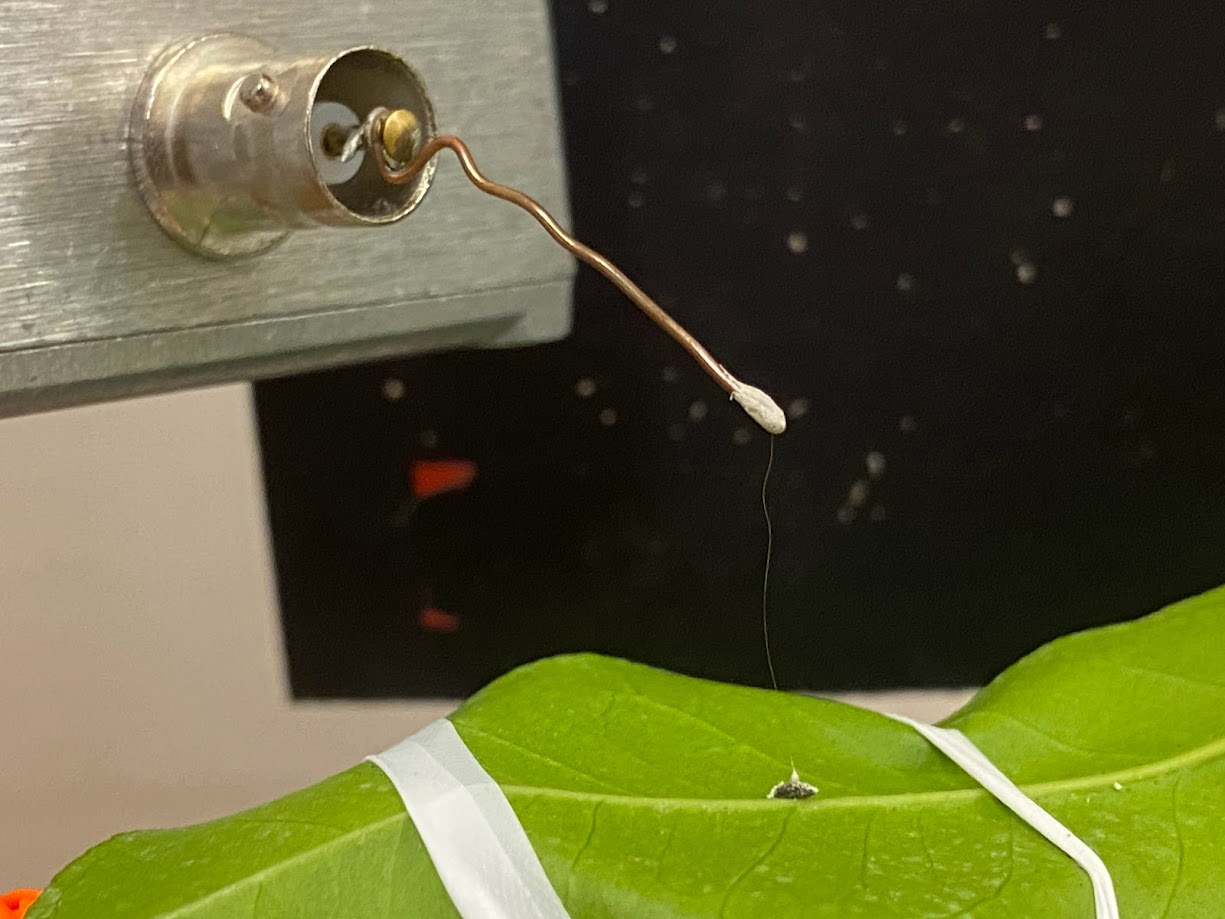


Head stage amplifier

25 µm Gold wire

**A**

Head stage amplifier

12 µm Gold wire

**B**

Mealybug


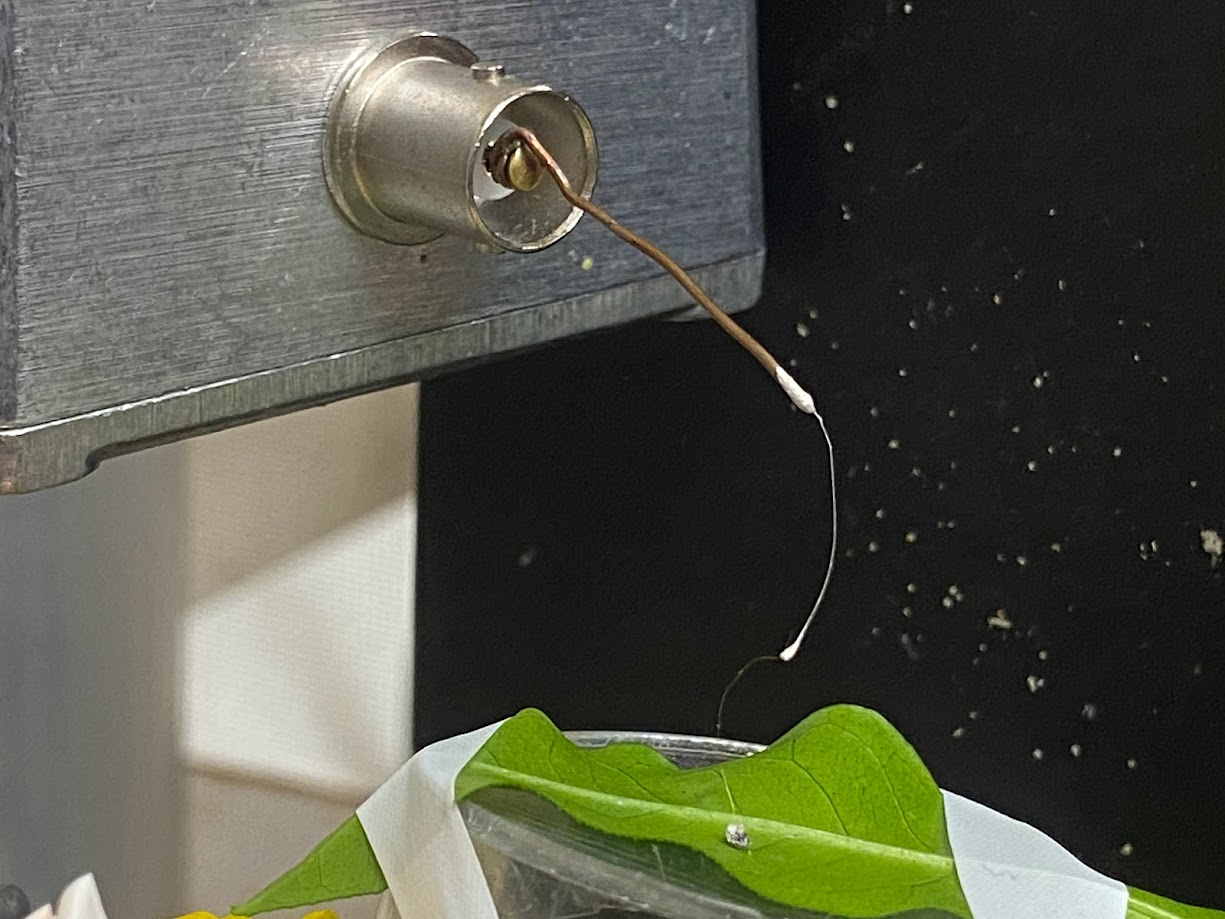


Head stage amplifier

Platinum wire

**C**

Copper wire

Fig. S2. Hibiscus mealybug, *Nipaecoccus viridis* with three tethering materials. A) 25 µm gold wire; B) 12.5 µm gold wire; C) Wollaston platinum wire.


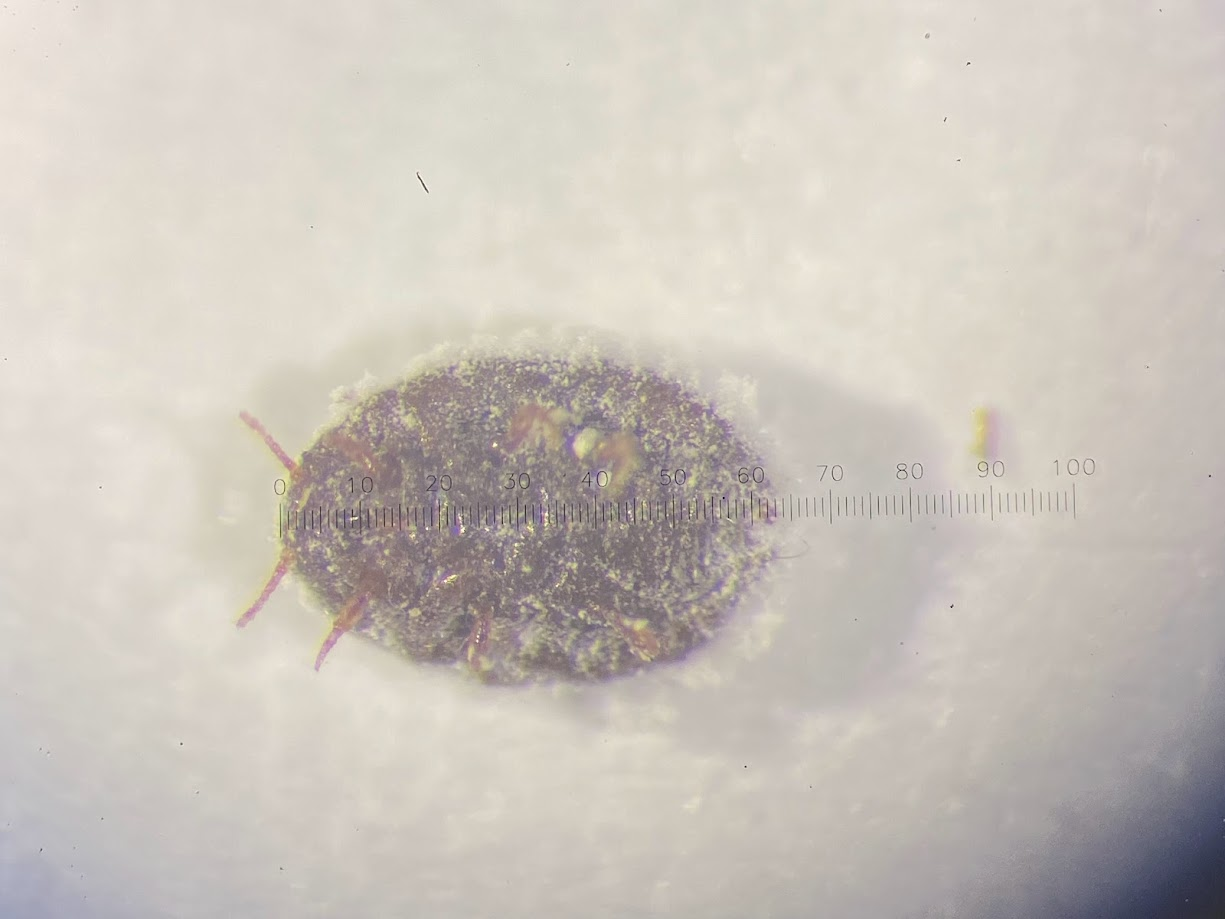

Fig. S3. Ventral view of a second instar of the hibiscus mealybug

1.0 mm

Table S1. Measurements of second-third instar hibiscus mealybug

| Mealybug # | 1 | 2 | 3 | 4 | 5 | 6 | 7 | Mean (µm) | Mean (mm) |
| --- | --- | --- | --- | --- | --- | --- | --- | --- | --- |
| Length (µm) | 2075 | 1775 | 2075 | 2000 | 1425 | 1625 | 1375 | 1764.29 | 1.76 |
| Width (µm) | 1450 | 1000 | 1500 | 1375 | 1050 | 1050 | 1200 | 1232.14 | 1.23 |


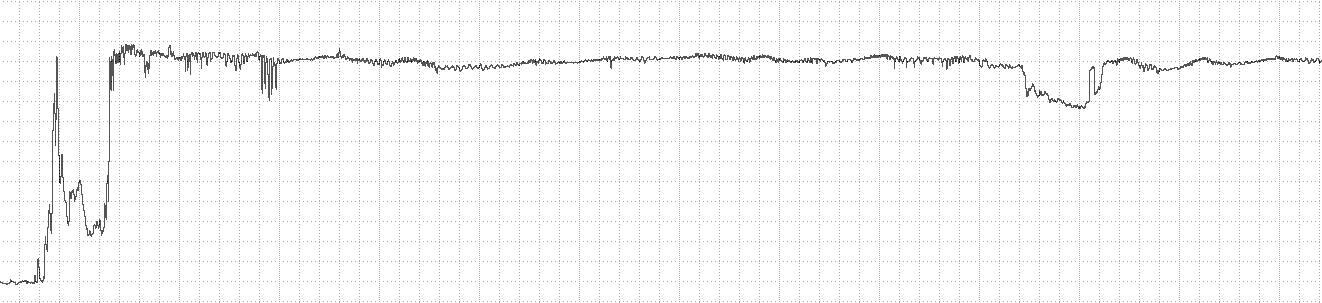


**10^10^Ω – 100 mV**


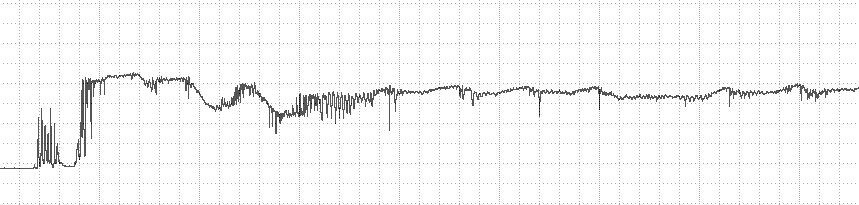


**10^9^Ω – 250 mV**

4× – 4.0 sec/div


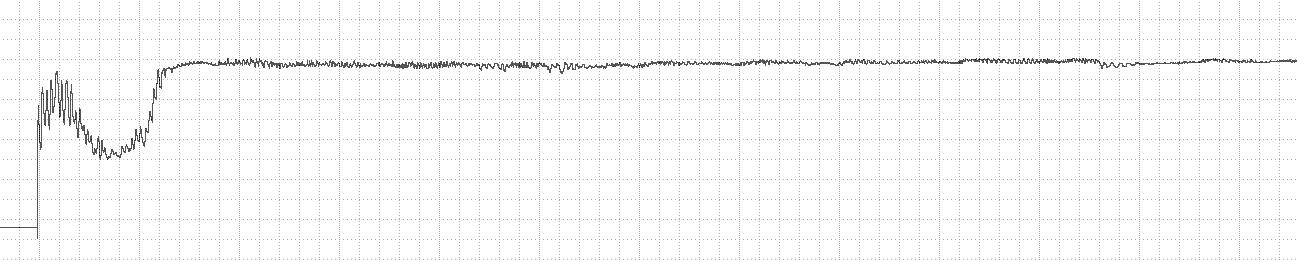


**10^13^Ω – 0 mV**

A

B

C

B

C

A

A

B

C

np

np

np

Fig. S4. Comparison of the beginning of a probe (waveforms A, B, and C) from *Nipaecoccus viridis* on *Citrus volkameriana* with 25 µm gold wire at three Ri level-substrate voltage combinations. Recordings were performed for 24 h with DC applied signal and Ri levels were switched during a recording (i.e. same insect). WinDaq gain and time scale are shown in the top box for 10^9^ Ω; the same gains and scales were used for the other boxes.


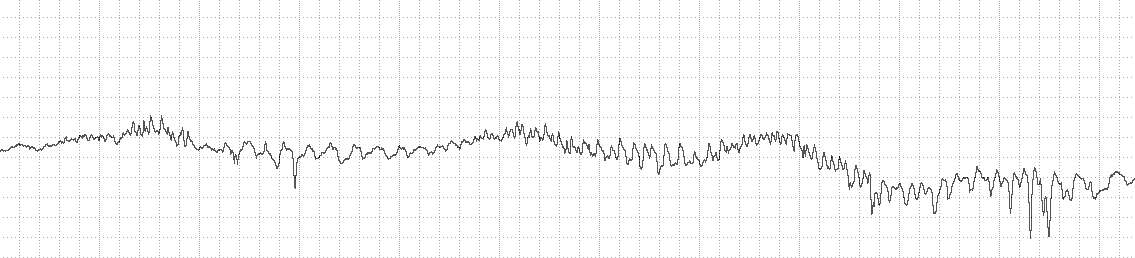


**10^10^Ω – 100 mV**


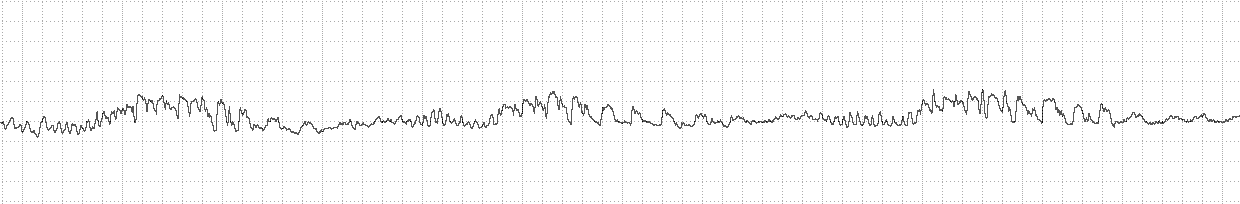

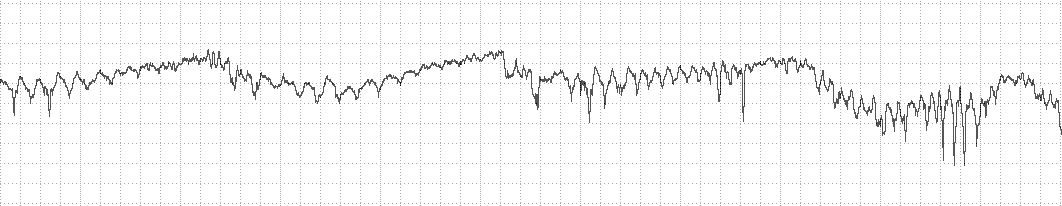


**10^9^Ω – 250 mV**

**10^13^Ω – 0 mV**

32× – 1.0 sec/div

Fig. S5. Comparison of the fine structure of waveform C generated by *Nipaecoccus viridis* on *Citrus volkameriana* with 25 µm gold wire at three Ri level-substrate voltage combinations (i.e. same insect). Recordings were performed for 24 h with DC applied signal and Ri levels were switched during a recording. WinDaq gain and time scale are shown in the top box for 10^9^ Ω; the same gains and scales were used for the other boxes.


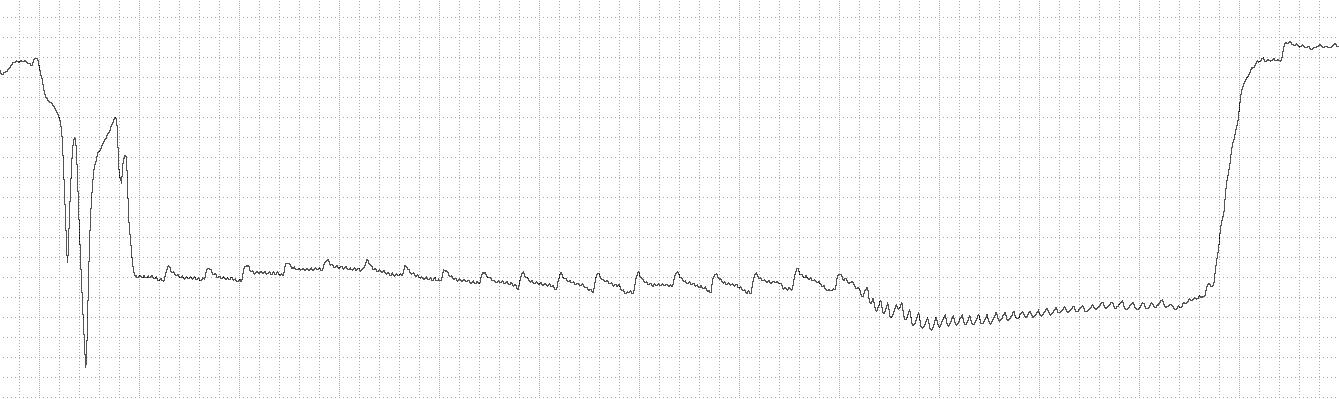

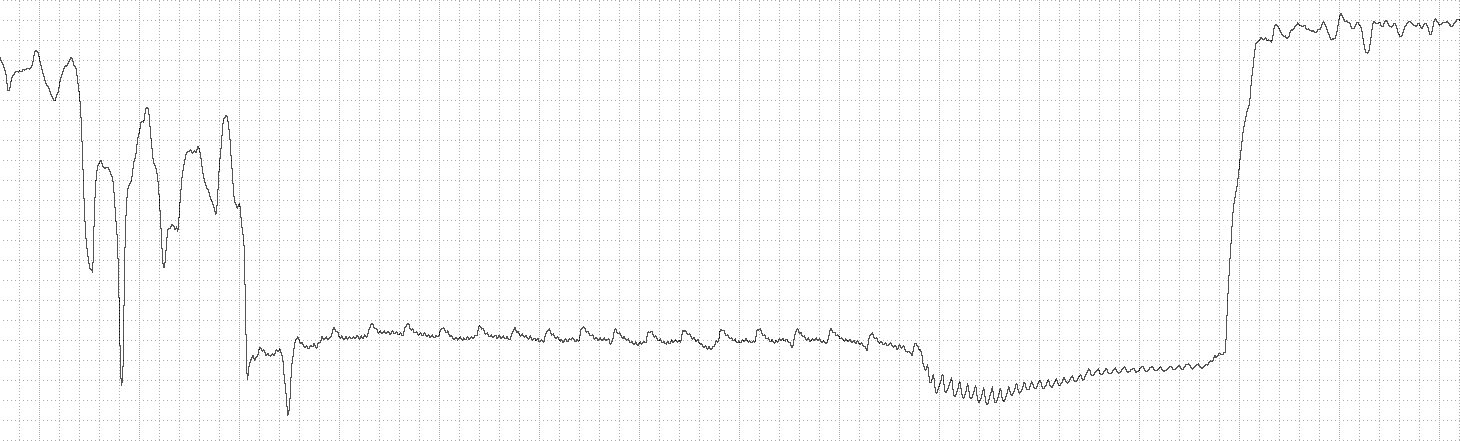

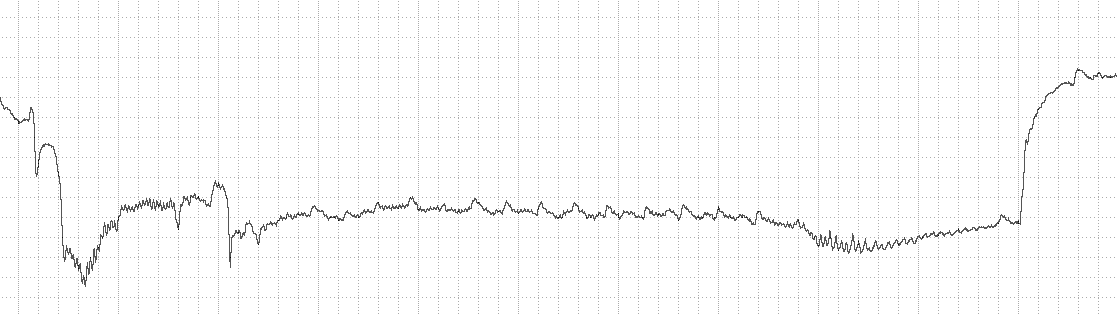


**10^10^Ω – 100 mV**

**10^9^Ω – 250 mV**

**10^13^Ω – 0 mV**

32× – 0.4 sec/div

Pre-pd

pd1

pd2

pd2

pd1

Pre-pd

pd1

pd2

Pre-pd

Fig. S6. Comparison of the fine structure of pd generated by *Nipaecoccus viridis* on *Citrus volkameriana* with 25 µm gold wire at three Ri level-substrate voltage combinations. Recordings were performed for 24 h with DC applied signal and Ri levels were switched during a recording (i.e. same insect). WinDaq gain and time scale are shown in the top box for 10^9^ Ω; the same gains and scales were used for the other boxes.


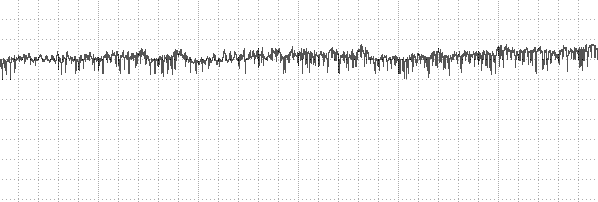


64× – 2 sec/div


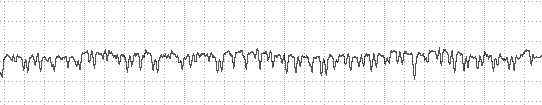


64× – 0.8 sec/div

**10^10^Ω – 100 mV**

Fig. S7. View of waveform E2 generated by *Nipaecoccus viridis* on *Citrus volkameriana* and showing characteristic downward peaks. Recordings were performed with 12 µm diameter gold wire for 24 h with DC applied signal. WinDaq gain and time scale are shown in the top right corner.


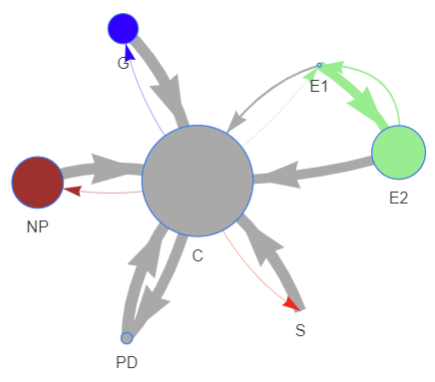


Fig. S8. Kinetogram of *Nipaecoccus viridis* when feeding on *Citrus volkameriana*. Colors were assigned manually; non-probing is brown, pathway is dark grey, xylem ingestion is blue, and phloem events are light green. Arrows are colored by their destination color. Proportional comparisons are valid.

*Notes: The kinetogram was generated using R (4.4.1 “Race for your life”, R Foundation for Statistical Computing) running in RStudio (2024.09.0 Build 375, Posit Software, PBC, Boston, MA). The package visNetwork (2.1.2 https://visjs.org/) was used to make the network plots referred to as kinetograms. The terminology in R differs significantly from the biological terminology. The package visNetwork requires input for nodes (behaviors), and edges (transitions). Therefore, the data was reprocessed to convert the behaviors to interpretable graphical objects. The nodes were the average proportion of time spent in that behavior relative to total recording time. This was converted to a diameter treating the duration as an “area.”* *The edges were scaled such that the width of arrows leaving a node total to 1. To make better plots all values were multiplied by 10.*
